# Evolutionary analysis of genes coding for Cysteine-RIch Secretory Proteins (CRISPs) in mammals

**DOI:** 10.1186/s12862-020-01632-5

**Published:** 2020-06-08

**Authors:** Lena Arévalo, Nicolás G. Brukman, Patricia S. Cuasnicú, Eduardo R. S. Roldan

**Affiliations:** 1 Department of Biodiversity and Evolutionary Biology, Museo Nacional de Ciencias Naturales (CSIC), c/José Gutiérrez Abascal 2, 28006 Madrid, Spain; 2 Institute of Pathology, Department of Developmental Pathology, University Hospital Bonn, Bonn, 53127 Germany; 3 Instituto de Biología y Medicina Experimental (IBYME-CONICET), C1428ADN Buenos Aires, Argentina

**Keywords:** Sexual selection, *CRISP*, Mammals, Sperm, Fertilization

## Abstract

**Background:**

Cysteine-RIch Secretory Proteins (CRISP) are expressed in the reproductive tract of mammalian males and are involved in fertilization and related processes. Due to their important role in sperm performance and sperm-egg interaction, these genes are likely to be exposed to strong selective pressures, including postcopulatory sexual selection and/or male-female coevolution. We here perform a comparative evolutionary analysis of *Crisp* genes in mammals. Currently, the nomenclature of *CRISP* genes is confusing, as a consequence of discrepancies between assignments of orthologs, particularly due to numbering of *CRISP* genes. This may generate problems when performing comparative evolutionary analyses of mammalian clades and species. To avoid such problems, we first carried out a study of possible orthologous relationships and putative origins of the known *CRISP* gene sequences. Furthermore, and with the aim to facilitate analyses, we here propose a different nomenclature for *CRISP* genes (*EVAC1–4*, “EVolutionarily-analyzed CRISP”) to be used in an evolutionary context.

**Results:**

We found differing selective pressures among *Crisp* genes. *CRISP1/4* (*EVAC1*) and *CRISP2* (*EVAC2*) orthologs are found across eutherian mammals and seem to be conserved in general, but show signs of positive selection in primate *CRISP1/4 (EVAC1).* Rodent *Crisp1* (*Evac*3a) seems to evolve under a comparatively more relaxed constraint with positive selection on codon sites. Finally, murine *Crisp3* (*Evac4*), which appears to be specific to the genus *Mus*, shows signs of possible positive selection. We further provide evidence for sexual selection on the sequence of one of these genes (*Crisp1/4*) that, unlike others, is thought to be exclusively expressed in male reproductive tissues.

**Conclusions:**

We found differing selective pressures among *CRISP* genes and sexual selection as a contributing factor in *CRISP1/4* gene sequence evolution. Our evolutionary analysis of this unique set of genes contributes to a better understanding of *Crisp* function in particular and the influence of sexual selection on reproductive mechanisms in general.

## Background

Proteins of the reproductive system that affect male and female traits are thought to be targets of accelerated gene sequence evolution [1, 2]. However, whereas this is generally true, evolutionary rates of reproductive proteins vary depending on their involvement in different reproductive processes or localization of expression [3–5]. One of the main driving forces promoting sequence divergence is postcopulatory sexual selection, either in the form of sperm competition or as cryptic female choice, which can additionally increase sexual conflict and drive male-female co-evolution leading to rapid adaptation. Sperm competition occurs when females mate promiscuously, and ejaculates of rival males compete for fertilizations. This leads to adaptations improving sperm performance; it may also intensify cryptic female choice and sexual conflict due to greater potential for selection [6].

The effect of postcopulatory sexual selection on evolutionary rates of reproductive proteins has been studied widely. Yet, it has been difficult to detect clear signals of sexual selection and only a small set of studies have found significant evidence by using correlational approaches. For example, the evolutionary rates of the coding sequences of seminal fluid proteins SEMG2 and SVS2, and of proteins expressed on the sperm surface (ADAM2 and ADAM18) and the acrosome (ZAN and SPAM1) have been found to be positively correlated with post-copulatory sexual selection in primates [7–11]. Other studies have reported negative correlations of sexual selection with evolutionary rates, such as in seminal fluid proteins in butterflies [12] and in sperm nuclear protamine 1 and protamine 2 in rodents [13–15]. Proteins found on the sperm surface [16] and those with roles in sperm motility and sperm-egg interaction [5] have been found to have particularly high evolutionary rates.

The mammalian spermatozoon is a very complex, polarized cell that needs to undergo a series of processes such as maturation in the epididymis and capacitation in the female tract in order to be able to reach, recognize and fertilize the egg in the oviduct. Spermatozoa carry numerous proteins involved in the acquisition of their fertilizing ability as well as in gamete interaction [3]. The Cysteine-RIch Secretory Protein (CRISP) family is of specific interest because it is involved in several of these processes and, therefore, is a likely target for sexual selection-driven evolution of gene sequences. Members of the CRISP family are mainly expressed in the mammalian male reproductive tract [17] and in the venoms of snakes [18]. Two major functional domains are present in all CRISPs: the PR-1 (or CAP) domain, which is thought to be involved in cell-cell adhesion, i.e., association between germ and Sertoli cells and sperm-egg fusion [17, 19, 20], and the Cysteine-Rich Domain (CRD), containing 16 conserved cysteine residues, with the capacity to regulate ion channels [17, 21]. The CRISP family, together with the Antigen-5 and the Pathogenesis related-1 proteins, form the CAP superfamily of proteins found in a wide range of organisms (bacteria, yeast, fungi, insects, plants and mammals, including human). The tertiary structure of CAP proteins shows a remarkable conservation despite often low overall identity and significant phylogenetic distance between organisms, suggesting that these proteins may be involved in common and essential biological processes [17]. A recent study investigating the evolutionary history of CAP proteins showed that the exon structure and borders of *CRISP* genes are remarkably conserved among vertebrates as compared to invertebrate CAP proteins [22].

Mammalian CRISPs are highly expressed during sperm cell development and maturation as well as during fertilization. Most mammals have three CRISP genes while in mice four CRISP members have been described: CRISP1, a mainly epididymal protein [23], CRISP2 [24], highly expressed in the testes, CRISP3 [25], which is widely distributed in reproductive and non reproductive organs, and CRISP4 which is mainly synthetized in the epididymis [26]. In the past few years, the use of in vitro approaches [20, 27–29] and knockout studies aimed at characterizing CRISPs [30–34] revealed the involvement of these proteins in different stages of the fertilization process (see review in [35]). Rodent CRISP1 binds to the sperm plasma membrane during epididymal maturation and is associated with both sperm-zona pellucida binding and gamete membrane fusion through the interaction of the protein with complementary sites in the egg [27, 36, 37]. Whereas evidence supports that the ability of CRISP1 to interact with the egg plasma membrane during gamete fusion resides in a region of only 12 amino acids within the PR-1 (CAP) domain that corresponds to one of the signatures of the CRISP family [20], the interaction of the protein with the zona pellucida does not reside in any specific region of the PR-1 domain but rather depends on the entire conformation of the molecule [29]. Interestingly, recent results showed that CRISP1 is also expressed in the cumulus cells that surround the egg and plays a role in fertilization [38]. Additionally, it has been revealed that CRISP1 has the ability to regulate CatSper [38], the principal sperm Ca^2+^ channel involved in the development of hyperactivation and essential for male fertility [39, 40]. Based on previous reports showing the ion regulatory activity of the CRD, it is likely that CRISP1 regulates CatSper through this domain [38]. Similar to rodent CRISP1, CRISP2 seems to play a role in gamete fusion [28, 29]. Recent knockout studies showing evidence for its involvement in hyperactivation development during capacitation and in both cumulus and zona pellucida penetration further strengthened this hypothesis [31]. Whereas no roles for CRISP3 in sperm function have been reported so far, the generation of CRISP4 knockout mice supports the involvement of this epididymal protein in fertilization [34, 36].

The sequences of mammalian *CRISP* genes were found to be conserved when compared to *CRISP* genes of snake venoms [41]. Yet, despite the prominent role of mammalian CRISPs in sperm capacitation and fertilization, comparative selective pressures and the role of sexual selection on CRISP gene sequence divergence has been scarcely addressed [5, 41, 42]. During this study, we aimed to fill this gap by examining patterns of selective pressures in mammalian clades and the role of sexual selection in the evolution of *CRISP* gene sequences. Currently, the nomenclature of the *CRISP* genes is somewhat confusing, as a consequence of discrepancies between assignments of orthologs, particularly due to numbering of *CRISP* genes [17], and this may generate problems when performing comparative evolutionary analyses between mammalian clades and species. Therefore, we first carried out an analysis of possible orthologous relationships and putative origins of the known *CRISP* gene sequences and, thus, propose a different nomenclature for *CRISP* genes when analyzed in an evolutionary context. Secondly, we analyzed selective pressures and sexual selection driving *CRISP* evolutionary rates for a relevant subset of *CRISP* genes. Even though *CRISP* gene sequences seem to be conserved in general [41], based on their involvement in sperm capacitation and sperm-egg interaction, we expected accelerated evolutionary rates concentrated on functionally relevant codon sites and regions. However, the main goal of this study was the general characterization of selection pressures (conservation, relaxation and/or positive selection) on *Crisp* gene sequences in mammalian clades. Additionally, we expected gene sequence divergence to be driven by sexual selection including sperm competition, cryptic female choice and/or male-female coevolution.

## Results

### *CRISP* nomenclature and sequence analysis

**Fig. 1:**
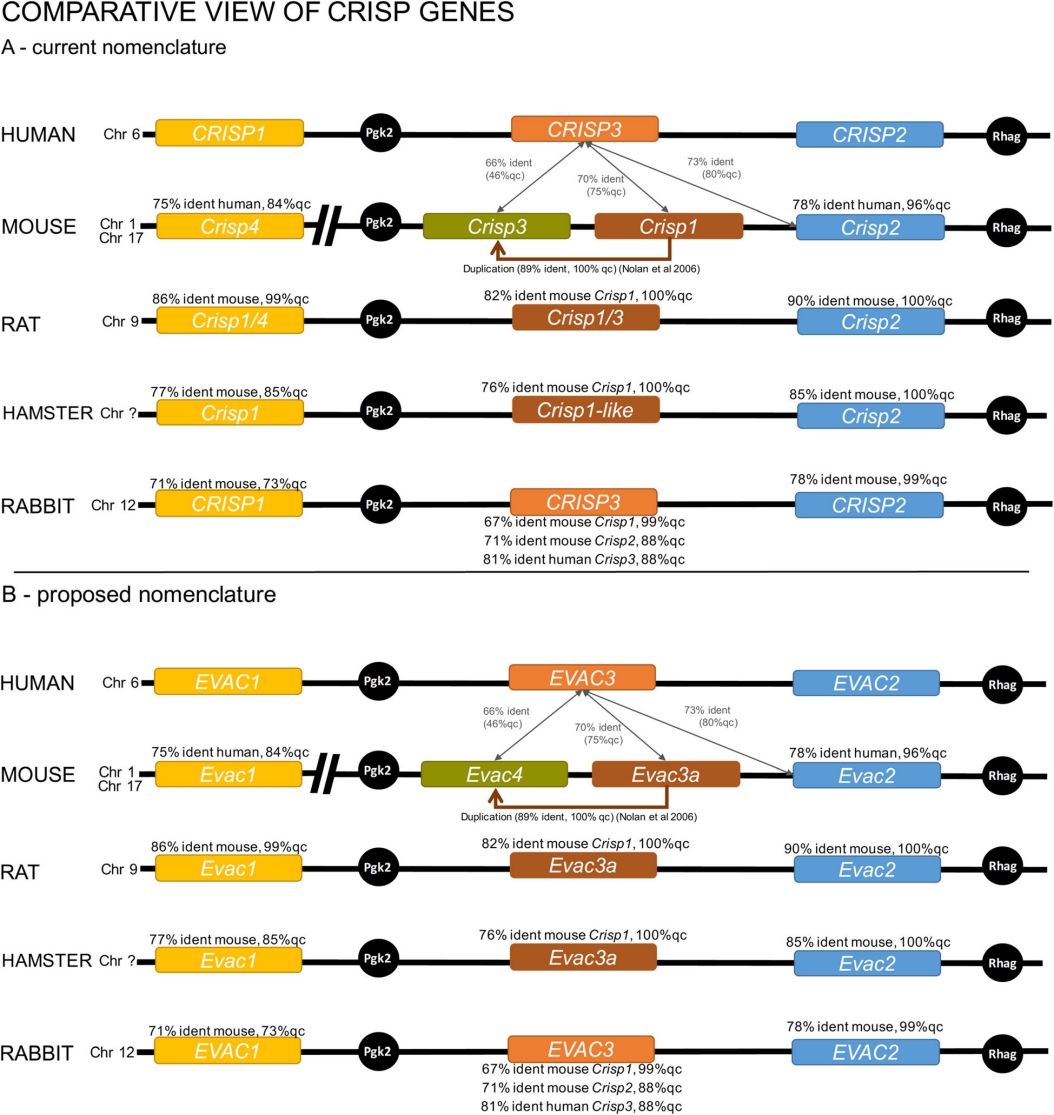
*CRISP* nomenclature and proposed evolutionary history. Information on chromosomal location, nomenclature and sequence identity of representative species is given. Sequence identity taken from NCBI nucleotide BLAST analyses using the genes coding sequences (Altschul et al. [43]), information available in GenBank databases and literature is included. (*A*) Current nomenclature. (*B*) Proposed nomenclature for comparative evolutionary analysis. Ident = sequence identity, qc = query cover. Coloring indicates orthology. In part modified from Vadnais et al. [44]

**Fig. 2:**
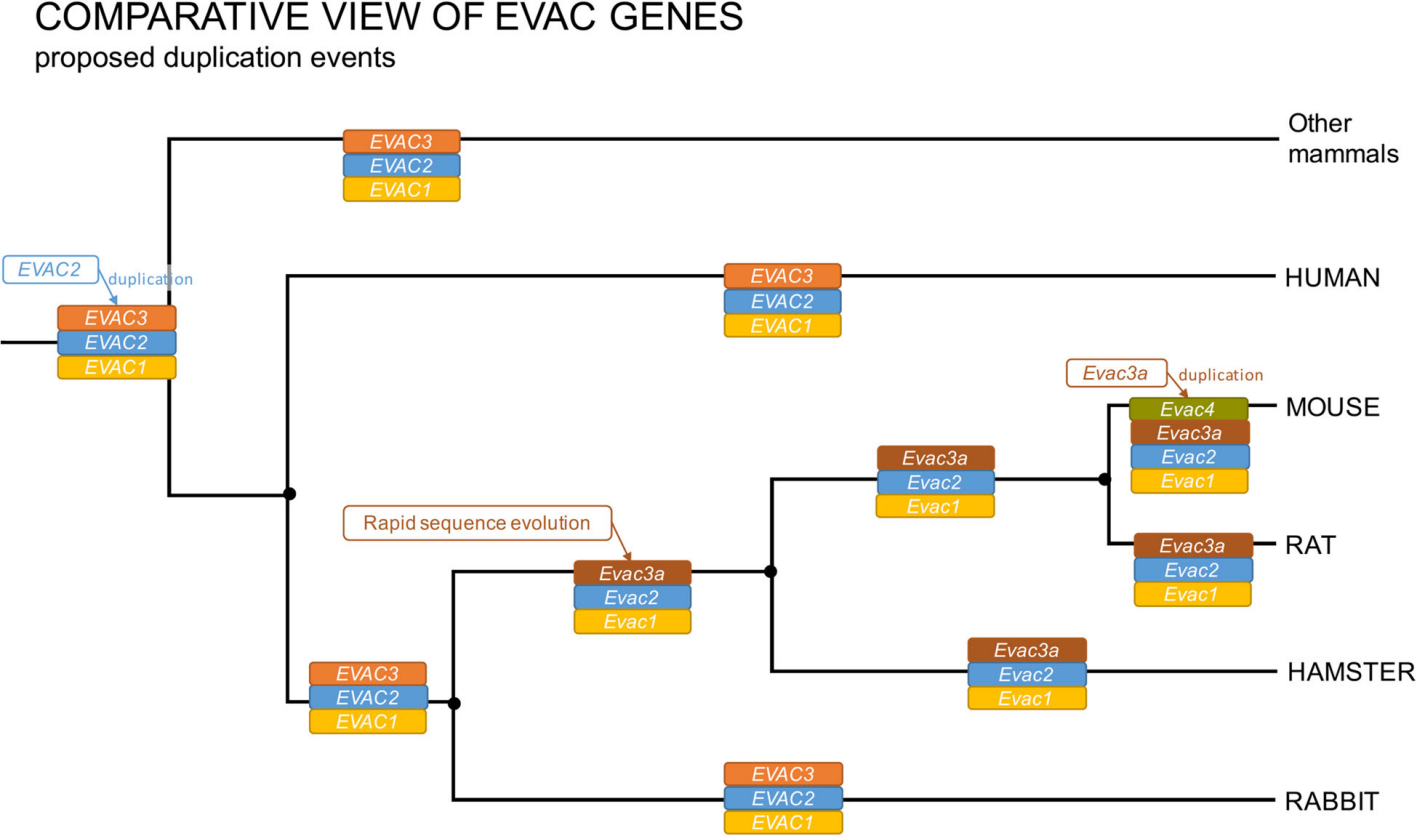
Proposed *CRISP* (*EVAC*) duplication events. Phylogeny according to Lüke et al. [14, 15]

In order to perform comparative evolutionary studies between clades and species for the different *CRISP* genes we first needed an analysis of sequence identity, location and possible origin of the different *CRISP* genes. Current nomenclature of *CRISP* genes can lead to confusion when comparing species and clades because of discrepancies in the numbering of *CRISP* genes (Fig. 1a). To avoid misinterpretations, we considered possible origins of *CRISP* genes during their evolutionary history based on gene sequence identity scores according to NCBI Blast [43], the existence or non-existence of specific *CRISP* orthologs in selected species, and information by Nolan et al. [45] and Vadnais et al. [44] (Fig. 1a). We also calculated a *CRISP* gene tree based on the sequence data used in this study (Additional files 1 and 2). Based on this information, we here propose a different nomenclature to be used in the evolutionary analyses of *CRISP* genes which are hereafter designed as “EVolutionarily-analyzed CRISPs” (*= EVAC*) (see Fig. 1b for details of nomenclature). In addition, we present a tentative representation of the origins of *EVAC* duplication events (Fig. 2). The numbering of *EVAC* genes employed here follows the proposed/possible sequence of evolutionary history from the most ancestral gene to the most recently arisen duplication. It should be borne in mind that this analysis is not exhaustive and has as its main goal attaining sufficient confidence in gene relationships so as to perform a comparative evolutionary analysis.

### Selective pressures *on EVAC1* and *EVAC2*

#### Across mammals

*EVAC1* and *EVAC2*, which are found across mammals (Fig. 1b), were tested for the general mode of selection acting upon them. To obtain the selective pressure acting on the whole sequence across all mammals, we calculated the evolutionary rate (ω) for the whole tree on the whole sequence (Codeml (PAML4) model M0, as explained in Materials and Methods). The evolutionary rate calculated across mammals in model M0 was *EVAC*1: ω = 0.48 and *EVAC*2: ω = 0.33.

#### Comparison of selective pressures between mammalian clades

Clade-specific analyses were performed on clades for which sequence data of at least 6 species were available (*EVAC1*: Primates, Rodentia, Carnivora, Cetartiodactyla; *EVAC2*: Primates, Rodentia, Chiroptera, Cetartiodactyla). To assess the comparative selective pressures for the entire sequence and selective pressures on codon sites, we employed branch analysis and branch-site analysis (see Materials and Methods), marking the clade of interest as foreground against the remaining species as background.

The branch analysis for *EVAC1* comparing clades suggests conserved selective constraint on all clades (LRT MCfixed vs MC significant, ω is significantly lower than 1) The selective constraint seems to be stronger in rodents and Cetartiodactyla (LRT M0 vs MC significant, MC ω considered, MC ω < M0 ω) (Table 1). The branch-site test for *EVAC1* showed positive selection on 2 codon sites for primates (BSfixed vs BS significant, 222-I, 235-I) (Table 1).

**Table 1:** Summary of results for *EVAC1* branch analysis and branchsite analysis for mammalian clades

***EVAC1***	LRTs for Branch Analysis		LRTs for BS analysis		Prop. of sites in ω classes (BS):					Interpretation	
Foreground branches	2Δ(M0-MC)	2Δ(MCfixed-MC)	2Δ(BSfixed-BS)	ω	0	1	2a	2b	PSS (BEB *p* < 0.05)	Selection over whole sequence	Selection on sites
Primates	−1.12	**−36.43**^**(< 0.01)**^	**−9.03**^**(0.04)**^	0.48	0.44	0.53	0.01	0.01	222 I, 235 I	conserved	positive
Rodentia	**−20.69**^**(< 0.01)**^	**− 152.48**^**(< 0.01)**^	0.00	0.35	0.44	0.53	0.01	0.01	–	conserved	no
Carnivora	−2.87	**−41.55**^**(< 0.01)**^	−5.97	0.48	0.45	0.55	0.00	0.01	–	conserved	no
Cetartiodactyla	**−20.90**^**(< 0.01)**^	**−98.98**^**(< 0.01)**^	0.01	0.29	0.45	0.55	0.00	0.00	–	conserved	no

Significant LRTs indicated in bold with FDR-corrected p-value in superscript, ω = clade omega as calculated by branch analysis, if LRT of M0 versus MC significant MC ω is reported if LRT non significant M0 ω is reported. ω site classes: 0: 0 < ω < 1 for foreground and background branches, 1: ω = 1 for foreground and background branches, 2a: 0 < ω < 1 for background and ω > 1 for foreground, 2b: ω =1 for background and ω > 1 for foreground. Applied models are explained in material and methods (MC = Clade model, BS = Branch-site model). *PSS* Positively selected sites, *BEB* Bayes empirical Bayes

The branch analysis for *EVAC2* also suggests conserved selective constraint on all clades (LRT MCfixed vs MC significant, ω is significantly lower than 1). Here, in Chiroptera a comparatively more relaxed constraint is detected (LRT M0 vs MC significant, MC ω considered, MC ω > M0 ω) (Table 2). The branch-site test for *EVAC2* showed no signs of positive selection on codon sites in either clade (BSfixed vs BS non significant) (Table 2).

**Table 2:** Summary of results for *EVAC2* branch analysis and branchsite analysis for mammalian clades

***EVAC2***	LRTs for Branch Analysis		LRTs for BS analysis		Prop. of sites in ω classes (BS):					Interpretation	
Foreground branches	2Δ(M0-MC)	2Δ(MCfixed-MC)	2Δ(BSfixed-BS)	ω	0	1	2a	2b	PSS (BEB *p* < 0.05)	Selection over whole sequence	Selection on sites
Primates	−1.59	**−63.97**^**(< 0.01)**^	0.02	0.33	0.61	0.39	0.00	0.00	–	conserved	no
Rodentia	−0.15	**−148.19**^**(< 0.01)**^	0.00	0.33	0.58	0.35	0.05	0.03	–	conserved	no
Chiroptera	**−13.57**^**(< 0.01)**^	**−5.47**^**(0.05)**^	0.00	0.63	0.61	0.39	0.00	0.00	–	conserved	no
Cetartiodactyla	−4.82	**− 113.47**^**(< 0.01)**^	1.27	0.33	0.61	0.39	0.00	0.00	–	conserved	no

Significant LRTs indicated in bold with FDR-corrected p-value in superscript, ω = clade omega as caculated by branch analysis, if LRT of M0 versus MC significant MC ω is reported if LRT non significant M0 ω is reported. ω site classes: 0: 0 < ω < 1 for foreground and background branches, 1: ω = 1 for foreground and background branches, 2a: 0 < ω < 1 for background and ω > 1 for foreground, 2b: ω =1 for background and ω > 1 for foreground. Applied models are explained in material and methods (MC = Clade model, BS = Branch-site model). *PSS* Positively selected sites, *BEB* Bayes empirical Bayes

### Selective pressures *on EVAC3*

#### Across rodents

*Evac3a* seems to be the ortholog of human *EVAC3* (Fig. 1b), although it seems to have evolved more rapidly in rodents leading to a lower sequence identity than expected when compared to non-rodent species or other *CRISP* family members (see Fig. 1a,b). We therefore confined our comparative analysis to rodent *Evac3a*. The alignment and phylogenetic tree used in evolutionary analyses is shown in additional files 4 and 5.

To obtain the selective pressure acting on *Evac3a* sequence across all rodents, we calculated the evolutionary rate (ω) for the whole tree (Codeml (PAML4) model M0 as explained in Materials and Methods). The evolutionary rate calculated across rodents in model M0 was ω = 0.53 (Table 3). We additionally performed a site-analysis to determine if specific codon sites are positively selected across rodents. Two sites show a trend towards positive selection (M7 vs M8 significant, M1a vs M2a non significant, 64-G, 73-T) (Table 3).

**Table 3:** Summary of results for *Evac3a* branch analysis, site and branchsite analysis for mammalian clades

***Evac3a***	LRTs for Branch Analysis		LRTs for BS analysis		Prop. of sites in ω classes (BS):					Interpretation	
Foreground branches	2Δ(M0-MC)	2Δ(MCfixed-MC)	2Δ(BSfixed-BS)	ω	0	1	2a	2b	PSS (BEB *p* < 0.05)	Selection over whole sequence	Selection on sites
*Cricetulus griseus*	−1.83	−0.38	−0.09	0.53	0.44	0.53	0.01	0.01	–	relaxed	no
*Marmota marmota*	**−7.18**^**(0.04)**^	−7.18	**−8.07**^**(0.03)**^	0.00	0.45	0.52	0.02	0.02	35 E, 161 Y	relaxed	positive
*Mesocricetus auratus*	−0.44	−4.51	0.00	0.53	0.46	0.54	0.00	0.00	–	conserved	no
*Microtus ochrogaster*	**−7.05**^**(0.04)**^	0.00	**−7.29**^**(0.03)**^	1.00	0.43	0.45	0.06	0.06	49 S, 225 K	relaxed	positive
*Mus musculus*	−0.12	−2.02	−1.67	0.53	0.45	0.54	0.00	0.01	–	relaxed	no
*Nannospalax galili*	−0.07	−3.69	**−7.30**^**(0.03)**^	0.53	0.43	0.53	0.02	0.02	(8 L)	relaxed	positive
*Peromyscus maniculatus*	0.00	−2.41	−5.03	0.53	0.45	0.54	0.01	0.01	187 S	relaxed	no
*Rattus norvegicus*	−0.05	−2.07	0.00	0.53	0.45	0.53	0.01	0.01	–	relaxed	no
	LRTs for Site Analysis				prop of sites in ω classes (M2a):						
Over all branches	2Δ(M7-M8)	2Δ(M1a-M2a)			0	1	2		PSS (BEB p < 0.05, both LRTs)		Selection on sites
Rodentia	**−7.54**^**(0.01)**^	−5.45			0.50	0.25	0.25		64 G, 73 T		positive (trend)

Significant LRTs indicated in bold with p-value in superscript (FDR-corrected for branch analysis and BS analysis), ω = clade omega as caculated by branch analysis, if LRT of M0 versus MC significant MC ω is reported if LRT non significant M0 ω is reported. ω site classes: 0: 0 < ω < 1 for foreground and background branches, 1: ω = 1 for foreground and background branches, 2a: 0 < ω < 1 for background and ω > 1 for foreground, 2b: ω =1 for background and ω > 1 for foreground. Applied models are explained in material and methods (MC = Clade model, BS = Branch-site model). *PSS* Positively selected sites, *BEB* Bayes empirical Bayes

#### Comparison of selective pressures between rodent species

Lineage-specific analyses were performed on species for which sequence data were available (*Cricetulus griseus, Marmota marmota, Mesocricetus auratus, Microtus ochrogaster, Mus musculus, Nannospalax galili, Peromyscus maniculatus, Rattus norvegicus*). Similar to the analysis of *EVAC1* and *EVAC2,* we assessed the comparative selective pressures for the entire sequence and on codon sites (branch analysis and the branch-site analysis), alternately marking a species branch as foreground against the remaining species as background.

The branch analysis for *Evac3a* comparing species suggests relaxed selective constraint (LRT MCfixed vs MC non significant, ω not significantly different from 1) on all species except *Mesocricetus auratus* for which a conserved constraint seems to be a better fit (LRT MCfixed vs MC significant, ω is significantly lower than 1) (Table 3). The branch-site test for *Evac*3a showed positive selection on codon sites for *Marmota marmota* (BSfixed vs BS significant, 35-E, 161-Y) and *Microtus ochrogaster* (BSfixed vs BS significant, 49-S, 225-K) (Table 3).

### Selective pressures *on Evac4*

**Fig. 3:**
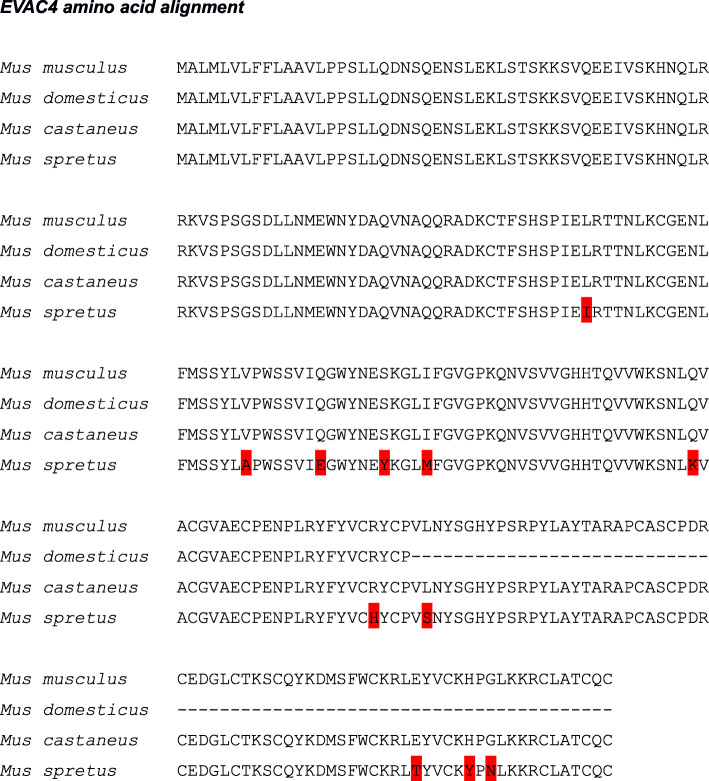
Amino acid alignment of *Evac4* sequences. Sequence data gathered via NCBI nucleotide BLAST of genome sequences from wild-derived mouse strains carried out by the Sanger sequencing project (Yalcin et al. [46]). Amino acid substitutions marked in red

Based on extensive research in GenBank and PhylomeDB databases and NCBI Blast analyses [43], we propose that the *Evac4* gene is a recent duplication present in the genus *Mus*. We did not expect any major differences in coding sequences between *Mus* species due to the high sequence identity between closely related species. In order to look for differences between species and sub-species in their coding sequence, we gathered *Evac4* gene sequences from the mouse wild-derived strain genome sequences available from the Sanger mouse genomes project [46]. Sequences from *Mus musculus musculus* strain PWK/PhJ, *Mus musculus domesticus* strain WSB/EiJ, *Mus musculus castaneus* strain CAST/EiJ, and *Mus spretus* strain SPRET/EiJ were trimmed to coding sequence and checked manually. The multiple sequence alignment was marked to visualize differences in coding sequence and amino acid changes. No differences were found between *Mus musculus* sub-species. A total of 14 nucleotide substitutions were found in the *Mus spretus* strain when compared to the *Mus musculus* strains. All except one of the nucleotide substitutions found in the *Mus spretus* sequence lead to amino acid changes (89 L > I, 107 V > A, 114 Q > E, 149 Q > K, 169 R > H, 174 L > S, 123 E > T, 230 G > N). It is therefore likely that this gene evolves under positive selective pressure. Not enough sequence divergence was found to reliably perform Codeml analysis or to test for effects of sexual selection (Fig. 3).

### Sexual selection on *EVACs*

**Fig. 4:**
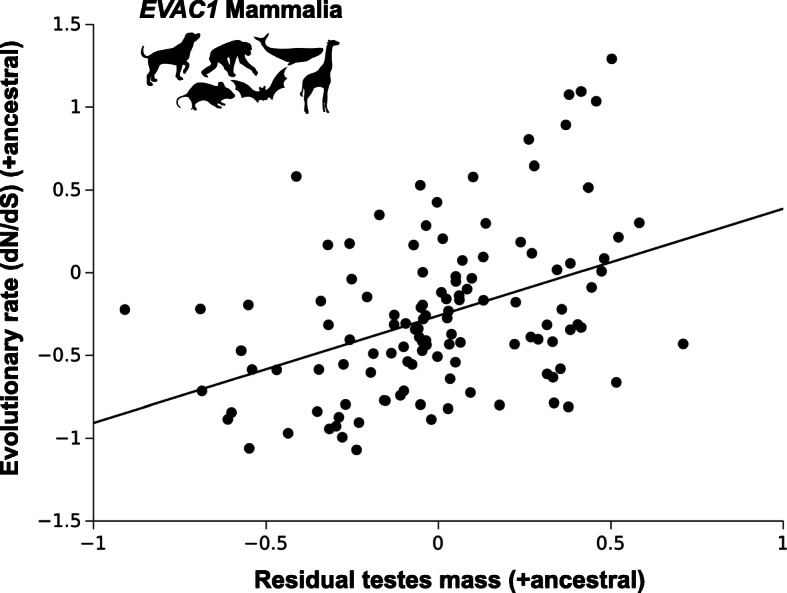
Relationship between *EVAC1* mammalian evolutionary rate (dN/dS) and residual testes mass including reconstructed ancestral node data. The association was examined by using COEVOL (see Table 4 for details)

To examine the possible effects of sexual selection on *EVAC* evolutionary rates we employed COEVOL using relative testes mass as proxy for postcolulatory sexual selection (see Methods) [47]. We first performed the analysis using all mammalian species. Then we analyzed each mammalian clade separately using a clade specific alignment and phylogenetic tree. Our results showed a trend for a correlation between relative testes mass and *EVAC1* ω in mammals (Fig. 4, Table 4). Within clades, we found a trend for a positive correlation between relative testes mass and *Evac1* ω in rodents (Table 4). Correlations for the remaining clades were not significant. No correlation was found between relative testes mass and *EVAC2* ω in mammals. Within clades, we found a trend for a positive correlation between relative testes mass and *EVAC*2 ω in Cetartiodactyla (Table 4). Correlations for the remaining clades were not significant. For *Evac3a* no significant correlations were found (Table 4).

**Table 4:** Results of COEVOL correlation analysis

	Clade	Variable 1	Variable 2	n	Covariances	Correlation coefficient	Posterior probability
***EVAC1***	**Mammalia**	**ω**	**relative testes mass**	**39**	**2.06**	**0.416**	**0.07**
	Carnivora	ω	relative testes mass	8	−1.29	−0.24	0.35
	Cetartiodactyla	ω	relative testes mass	9	2.29	0.45	0.17
	**Rodentia**	**ω**	**relative testes mass**	**6**	**3.53**	**0.65**	**0.09**
	Primates	ω	relative testes mass	11	2.40	0.41	0.19
***EVAC2***	Mammalia	ω	relative testes mass	39	1.21	0.35	0.14
	**Cetartiodactyla**	**ω**	**relative testes mass**	**10**	**0.72**	**0.67**	**0.06**
	Rodentia	ω	relative testes mass	7	−1.55	−0.51	0.17
	Primates	ω	relative testes mass	10	−0.16	− 0.11	0.40
***Evac3a***	Rodentia	ω	relative testes mass	6	0.06	0.09	0.58

Correlations with relative testes mass are corrected for body mass (see material and methods section), ω = evolutionary rate computed by COEVOL, *n* = number of species in analysis, trends are shown in boldface

## Discussion

In this study we present an overview of selective pressures and tendencies of sexual selection-driven evolution of *CRISP* gene sequences. Moreover, due to differences in *CRISP* nomenclature between species we here propose a different set of gene names for use in evolutionary studies of *CRISP* (Fig. 1). Previous work has dealt with this inconsistency but a clear consensus of relationships between *CRISP* genes is not yet available [17, 44]. Based on our analysis, we propose that mouse *Crisp1* (*Evac3a*) is an ortholog of human *CRISP3* (*EVAC3*) that appears to have undergone rapid sequence divergence within the rodent clade after a putative duplication from *Crisp2* (*Evac2*). We base this proposal on comparisons of sequence identities and gene tree clustering which show that human *EVAC3* clusters more closely with mouse *Evac2*, the gene from which mouse *Evac3a* probably derived, rather than with mouse *Evac3a*. Additionally, rabbit *EVAC3* shows higher sequence identity with human *EVAC3* than with mouse *Evac3a*. Human *EVAC3* (human *CRISP3*) and mouse *Evac4* (mouse *Crisp3*) have so far been assumed to be orthologs, mostly based on the fact that both genes show a wider range of expression compared to other *EVAC* genes. We could not find strong evidence for this. The sequence identity and clustering in the gene tree provide more evidence for a recent mouse-specific duplication event, with *Evac4* deriving from *Evac3a* in mice. Previous studies have already shown *CRISP3* (human *EVAC3*, mouse *Evac4*) to be ambiguous. Vadnais et al. [44] reported that the GenBank sequence for pig *CRISP3* was *CRISP2*. Pig *CRISP3* has then been found within an unannotated region. *CRISP3* sequence for pig, horse and cow cluster together but differ from human and mouse *CRISP3* [44]. The diversity we see in the mammalian *CRISP* gene family and the difficulty resolving the orthologous relationships might be explained by rapid divergence of the gene cluster itself and, possibly, an increased susceptibility for duplication events. This certainly warrants further studies. The conclusions drawn here can only be considered preliminary and this analysis has been done for the sole purpose of gaining sufficient confidence in the understanding of interspecific relationships to undertake this comparative evolutionary study. A detailed analysis of the *CRISP* gene relationships and an adjustment of the nomenclature is necessary and is of great importance for future studies and to avoid wrong conclusions.

In this study we found *EVAC1* and *EVAC2*, which are found across eutherian mammals, to be conserved in general, with signs of positive selection in primate *EVAC1*. *Evac*3a seems to evolve under a comparatively more relaxed constraint with positive selection on codon sites consistent with its proposed rapid divergence in the rodent clade. According to our findings, *Evac4* seems to be specific to the genus *Mus* and shows signs of possible positive selection. Sexual selection seems to play a role in *EVAC* evolution and generally seems to favor an increase in evolutionary rate, although none of these trends have been found to be statistically significant.

EVAC proteins have been shown to be involved in various stages of the fertilization process, such as zona pellucida binding and gamete fusion [35]. Therefore, male expressed EVACs might show signs of co-evolution with female specific interaction partners. In fact, previous studies have provided evidence for a possible co-evolution, and therefore interaction, between EVAC1 and EVAC2 and egg cell membrane protein CD9 in primates [42]. Signals of positive selection concentrated on specific regions of the gene sequence might thus be an indication of co-evolution with a binding partner. In the case of rodent *Evac*3a, there are positively selected sites found in the PR-1 (CAP) domain across rodents as well as in the CRD domain in several species. Positively selected sites in the PR-1 (CAP) domain of *Evac3a* are of specific interest here since it has been shown that this domain might be involved in gamete fusion [20], making it a very likely target for co-evolution with female binding partners. We also found positively selected sites in primate *EVAC1*, here located in the CRD region, which is likely to be involved in the regulation of ion channels such as CatSper [21, 38]. Adaptive evolution in its sequence might lead to a more efficient regulation or adjustment to different types of ion channels. An analysis of co-evolution between CRISP genes and possible (female) binding partners such as CD9, in a wider range of species would be of interest.

*EVAC1* shows the strongest evidence for sexual selection-driven sequence evolution across mammals. *EVAC1* is expressed mainly in the epididymis and is involved in sperm capacitation and sperm-egg interaction in cooperation with other EVACs [35]. Interestingly, this gene seems to be the only EVAC not found in female reproductive tissues [48, 49] which may explain why a trend for a correlation with relative testes mass, a proxy for sperm competition, was found in this gene while not in the others. Genes acting in both male and female reproductive tissues might be subjected to different, even opposite pressures, due to sexual conflict, possibly obscuring any detectable signal of sexual selection during analysis. In genes confined to expression in male reproductive tissues, selective pressures due to sexual selection are more straightforward following only one direction, thus improving detection.

## Conclusions

Even though *EVAC1* and *EVAC2* both seem to be conserved in general, which might be explained by their potential additional roles in other processes [17], positive selection can still be a factor in the evolution of these two genes. Positive selection might be detectable on lower taxonomic levels or might have happened in intervals during the genes evolutionary history. Similarly, the lack of a strong signal of sexual selection does not preclude a role for this selective force on *EVAC* sequence evolution. Selective pressures might not focus solely on gene sequence but may act on a larger scale, i.e., on regulatory sequences or favoring duplication events, as shown for Zonadhesin, a sperm ligand involved in sperm-egg interaction [50]. This might be especially true in mice which, so far, are the only mammals known to express four *Crisp* genes. Additionally, as shown in previous studies, sexual selection might be harder to detect across a wide range of clades since these pressures might affect taxa differently [14, 15]. This might be the case in *EVAC2* where we found signs of sexual selection acting only on cetartiodactylan sequences. An analysis testing for associations of regulatory sequences, epigenetic marks, number of gene duplication events, and transcript variants with levels of female promiscuity would be of great interest for this protein family. Although, the assignment of orthologs and the nomenclature need to be completely resolved in order to address future studies, we believe our observations contribute to a better understanding of CRISP family evolutionary history.

## Methods

### Sequence data and phylogenetic tree

Gene sequences of mammalian CRISPs (here called EVACs, as explained in Results and Discussion, and including the following: for *EVAC1*, 61 species; for *EVAC2*, 65 species; for *Evac3a*, 8 rodent species; and for *Evac4*, 4 mouse species) were obtained from NCBI GenBank (Additional file 3), visualized with Geneious 5.5.9 (Biomatters, http://www.geneious.com/) and trimmed to coding sequences based on NCBI GenBank information. Sequences were manually checked to ensure correct trimming. Translation alignments were performed with PRANK [51] and subsequently stripped of columns containing gaps in more than 50% of the species to avoid bias due to ambiguously aligned regions [52]. PRANK is a phylogeny-aware progressive alignment especially applicable to analysis of selective pressures on coding sequences [53].

For *EVAC1* and *2,* in addition to alignments including all mammalian species (Additional files 4 and 5), we performed separate alignments for each mammalian clade studied (Primates, Rodentia, Chiroptera, Carnivora, Cetartiodactyla).

The phylogenetic trees of species included in this study were constructed as a consensus of phylogenies available from the literature (Additional files 6 and 7 and references therein).

### Preliminary analysis of orthologous relationships between CRISP genes

We determined potential orthology based on gene sequence identity scores according to NCBI Blast [43], the existence or non-existence of specific *CRISP* orthologs in selected species, their genomic location and information by Nolan et al. [45] and Vadnais et al. [44]. Relationships were further investigated using PhylomeDB (http://phylomedb.org; 28-July-2017), a database of gene phylogenies providing information about the evolutionary history of genes by visualization of multiple sequence alignments and phylogenetic trees.

In addition to this, and using the method described above, we produced an alignment of all *CRISP* gene sequences included in this study, which was then used to calculate a *CRISP* gene tree. The gene tree was constructed using RAxML, implemented in Geneious 5.5.9 (Biomatters, http://www.geneious.com/), with 100 replicates of rapid bootstrapping.

### Analysis of selective pressures

The nonsynonymous/synonymous substitutions rate ratio (ω, dN/dS or evolutionary rate) is an indicator of selective pressure at the protein level, with ω = 1 indicating neutral evolution, ω < 1 purifying selection, and ω > 1 diversifying positive selection [54]. To estimate gene sequence evolutionary rate across all mammals and additionally within mammalian clades, we used the application Codeml implemented in PAML 4 [55, 56]. Codeml calculates the evolutionary rate based on different models. It takes as input a multiple sequence alignment and the corresponding phylogenetic tree. It then estimates evolutionary rates for the whole tree, each branch or branch groups, taking into account either the whole sequence, or each codon separately. The Codeml models applied are explained below. Likelihood-ratio-tests (LRT) were performed to test if the alternative model presents a better fit to the dataset against the null model. For the Codeml codon frequency setting, as well as for the number of categories, we used the setting with the best fit for each analysis according to the preliminary likelihood-ratio-analysis.

### Evolutionary models applied in Codeml (PAML4)

#### Branch analysis

In order to obtain the evolutionary rates of mammalian clades, we computed the clade model comparing marked foreground branches (clade of interest) against the unmarked background in the analyzed phylogenetic tree. Three models were computed: M0 “one ratio” in which all branches were constrained to evolve at the same rate; MCfixed “two-ratio, foreground fixed” where the background ω was allowed to be estimated freely while the foreground ω was restrained to a value of ω = 1; and MC “two ratio” model which estimates for both the background and the foreground clade a free and independent ω. To test if the foreground evolves at a significantly different rate than the background, we compared M0 versus MC by means of LRT. If foreground ω was significantly higher than 1 (LRT significant for MCfixed vs MC and ω > 1) we assumed positive selection acting on the foreground branches on whole sequence level. If foreground ω was significantly lower than 1 (LRT significant for MCfixed vs MC and ω > 1) we report purifying selection acting on the branch on whole sequence level. Relaxed selective constraint for the foreground branch is assumed if foreground evolves at a significantly different ω than the background (M0 vs MC), and this ω was not significantly different from 1 (MCfixed vs MC) [57]. *P*-values of LRTs were false discovery rate (FDR)-corrected.

#### Branch-site analysis

Similarly, two models were computed to test evolution along coding sequences and infer codons under positive selection for marked foreground branches (clade of interest) in contrast to the unmarked background. BSfixed “branch-site model A, foreground fixed” in which the codon site ω for background branches is allowed to be computed freely while the foreground is fixed and BS “branch-site model A” in which codon sites in both foreground and background are computed freely [58]. If LRT between BSfixed and BS is significant, and sites significantly belonging to the positive selected codon site (PSS) category are detected, we report positive selection on the detected codon sites for the clade of interest. P-values of LRTs were FDR-corrected.

#### Site-analysis

To apply a test for positive selection on codon sites across all branches, which is of interest in case of rodent *Evac3a*, we applied a LRT comparing a null model that does not allow sites with ω > 1 with an alternative model that does. We applied two LRTs that have been widely used for this approach. The first compared model M1a “nearly neutral”, which assumes values for ω between 0 and 1, with model M2a “positive selection” which allows values of ω > 1. The second test compares two models assuming a β distribution for ω values. In this case, the null model M7 that limits ω between 0 and 1 is compared to the alternative model M8, that adds an extra class of sites with an ω ratio estimated that can be greater than 1 [59, 60]. We report positive selection on codon sites if LRT between both models is significant and sites significantly belonging to the positive selected site category are detected. If only one LRT is significant, we report a trend for the existence of PSS. Only sites significantly belonging to the positive selection site category in both alternative models are reported.

### Association between evolutionary rate and relative testes mass

Sperm competition, evoked by females mating promiscuously, is a powerful selective force. An almost universal response to increased levels of sperm competition is an increase in testes size and sperm production [61]. The relationship between increased levels of sperm competition and larger relative testes mass has been widely demonstrated [62] and has been shown to associate to genetic paternity [63]. Thus, relative testes mass has been commonly used as a proxy for levels of sperm competition and female promiscuity. We here use relative testes mass as proxy for female promiscuity and increased selective pressure due to sexual selection in general since many CRISPs are sperm surface proteins and have the potential to be affected by sperm competition, cryptic female choice or male-female co-evolution. Data on both body and testes mass were obtained from the literature (Additional file 3). Residual testes mass data were obtained from a regression analysis including body mass as independent and testes mass as dependent variables, and used for graphical representation of multiple regression results.

To test for an association between evolutionary rate of *EVAC* gene sequences and sexual selection, we employed the program COEVOL. COEVOL is a Bayesian Markov Chain Monte Carlo sampling software. This approach is used to test for correlation between genotype and phenotype data. It allows for a joint estimation of evolutionary rates for the input alignment and changes in the phenotypic input variables. Importantly, this software allows for detection of associations between genotypic and phenotypic data taking into account estimates of ancestral nodes, compared to previous approaches whereby the evolutionary rate was averaged from the root to the tip [47]. To test for sexual selection, correlations between testes mass and evolutionary rate were corrected for body mass by COEVOL using a multiple regression approach.

## Supplementary information

**Additional file 1.** CRISP gene tree (RAxML).

**Additional file 2.** PhylomeDB tree of seeding on mouse CRISP3 (EVAC4).

**Additional file 3.** Phenotype and gene sequence accession data.

**Additional file 4.** Crisp1/4 (Evac1) gene sequence alignment.

**Additional file 5.** Crisp2 (Evac2) gene sequence alignment.

**Additional file 6.** Crisp3 (Evac3a) gene sequence alignment.

**Additional file 7.** Phylogenetic trees of species included in the analysis for A-Evac1 and B-Evac2.

**Additional file 8.** Phylogenetic trees of species included in the analysis for Evac3.

## Data Availability

All data generated or analysed during this study are included in this published article. All gene sequences were taken from public databases (see details in Additional file 3). All phenotypic data (body mass and testes mass) were taken from the literature (Additional file 3).
