# Supplementary figures and images for: Evolutionary analysis of genes coding for Cysteine-RIch Secretory Proteins (CRISPs) in mammals
Source: BMC Evol Biol. 2020 Jun 8;20:67. doi: 10.1186/s12862-020-01632-5 (PMC7278046; doi:10.1186/s12862-020-01632-5)

## Additional file 1. CRISP gene tree (RAxML)

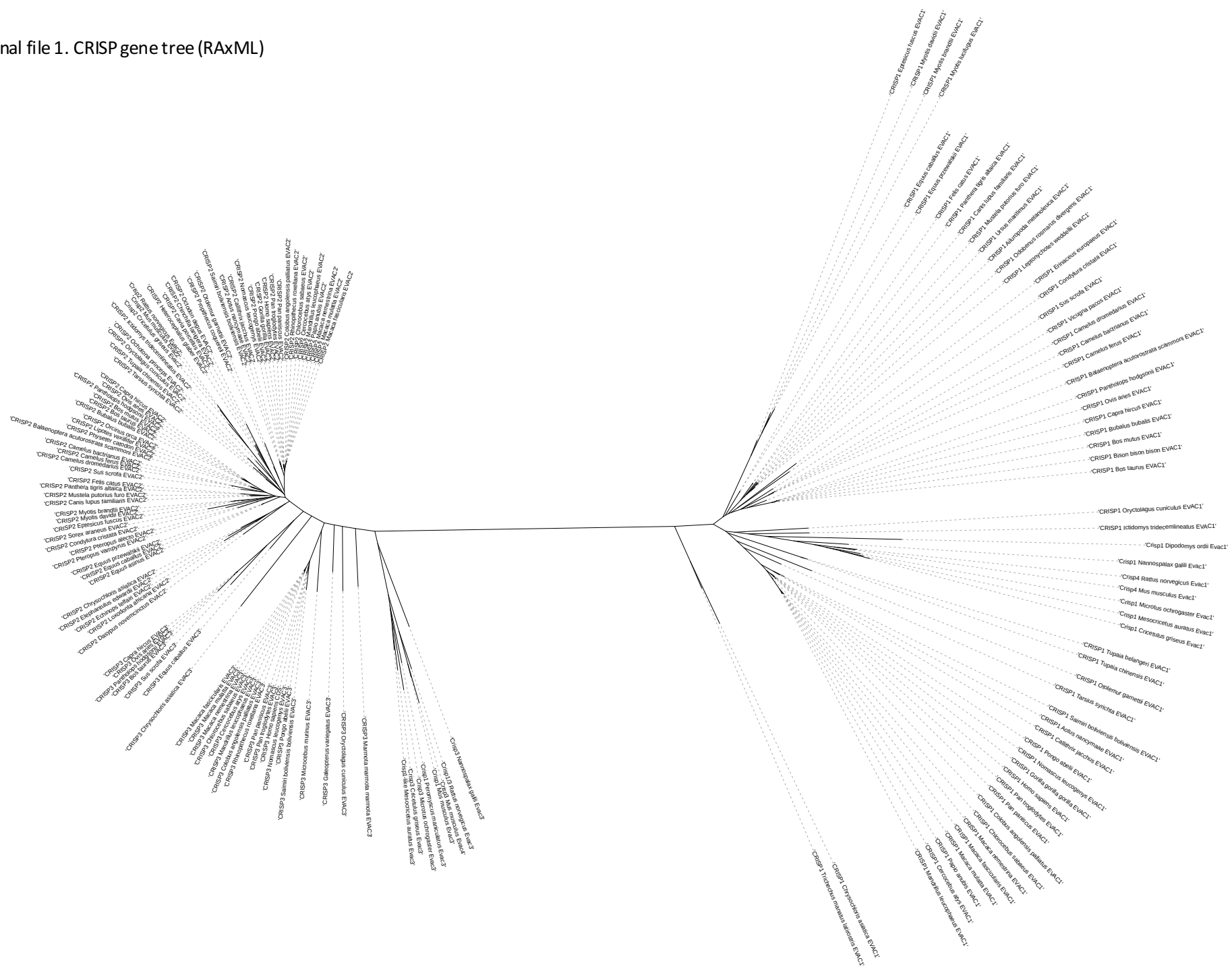

Supplement: Supplementary file 1 — Additional file 1. CRISP gene tree (RAxML). [file 12862_2020_1632_MOESM1_ESM.pdf]

Additional file 4. *Crisp1/4 (Evac1)* gene sequence alignment.

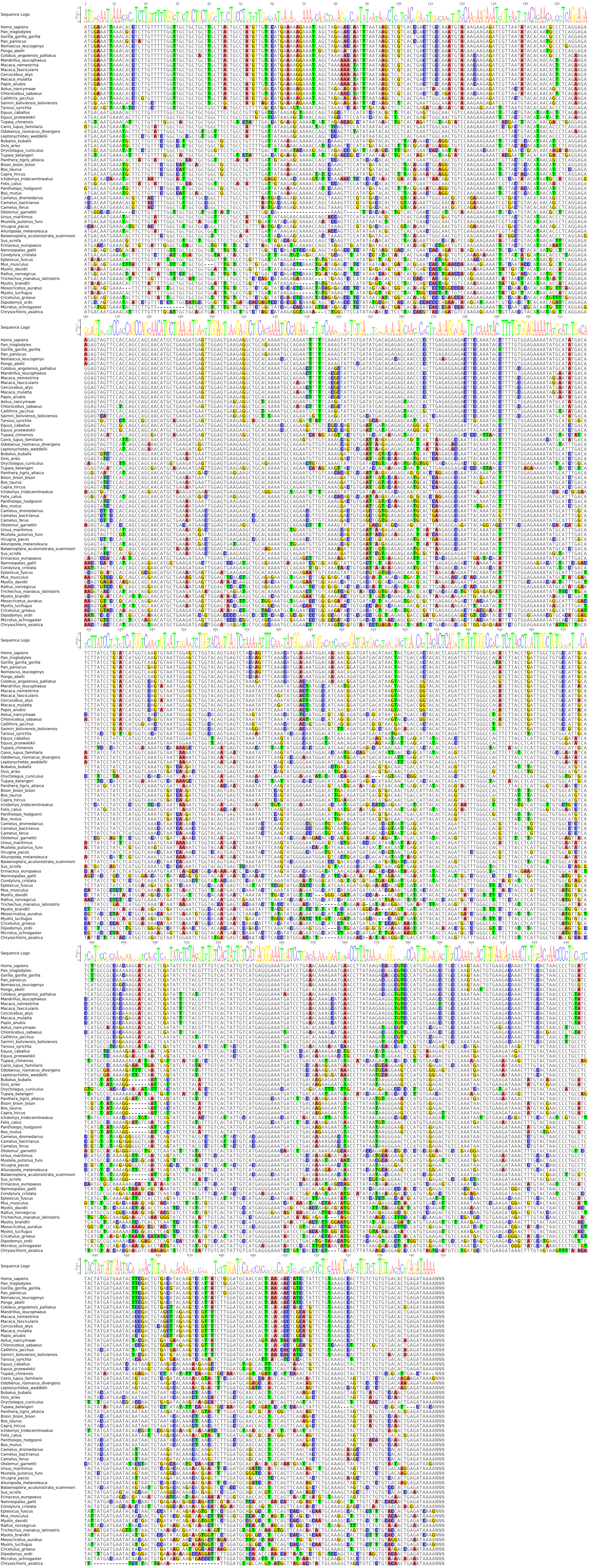

Supplement: Supplementary file 4 — Additional file 4. Crisp1/4 (Evac1) gene sequence alignment. [file 12862_2020_1632_MOESM4_ESM.pdf]

Additional file 5. *Crisp2 (Evac2)* gene sequence alignment.

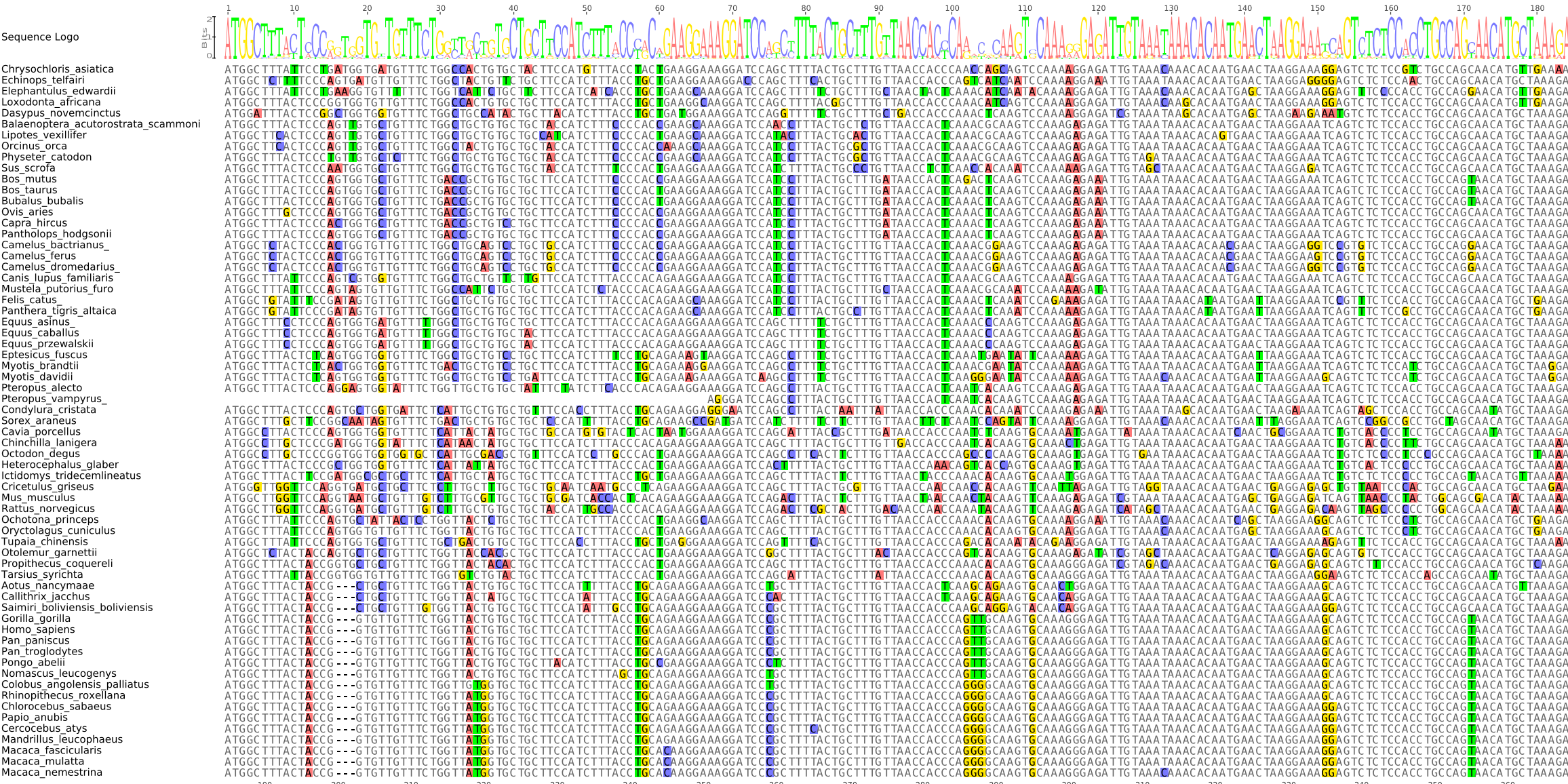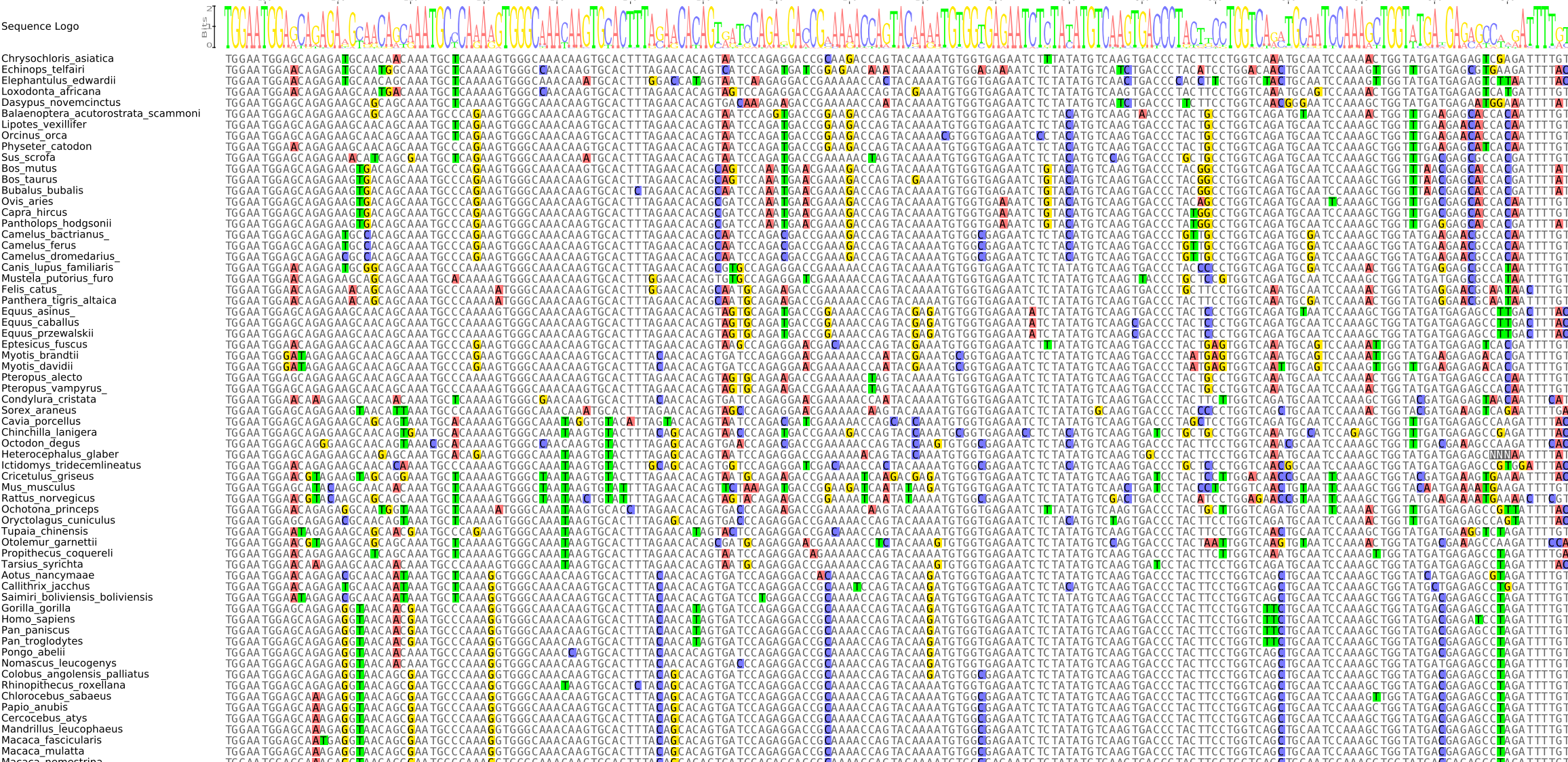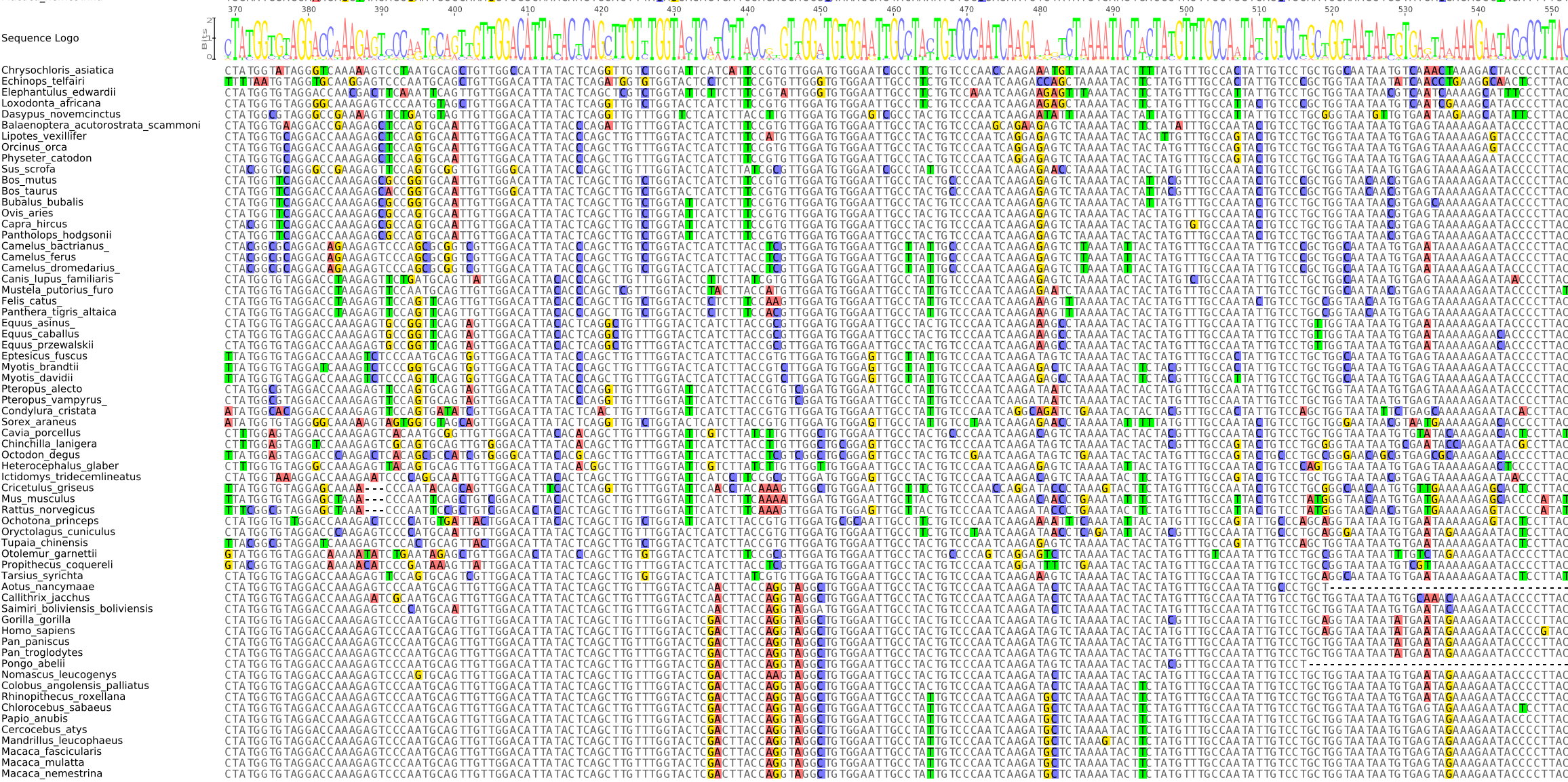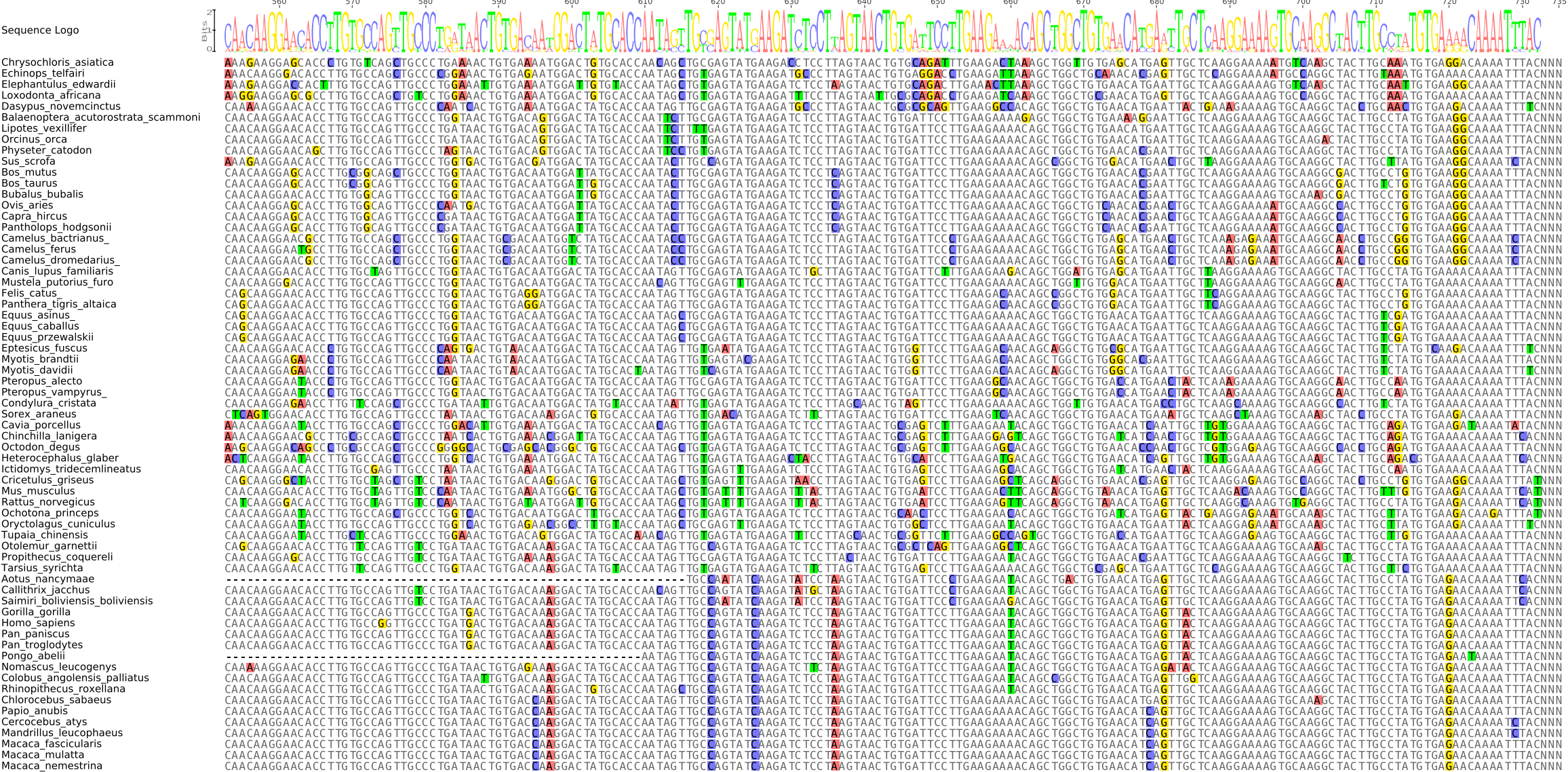

Supplement: Supplementary file 5 — Additional file 5. Crisp2 (Evac2) gene sequence alignment. [file 12862_2020_1632_MOESM5_ESM.pdf]

## Additional file 6. *Crisp3 (Evac3a)* gene sequence alignment.

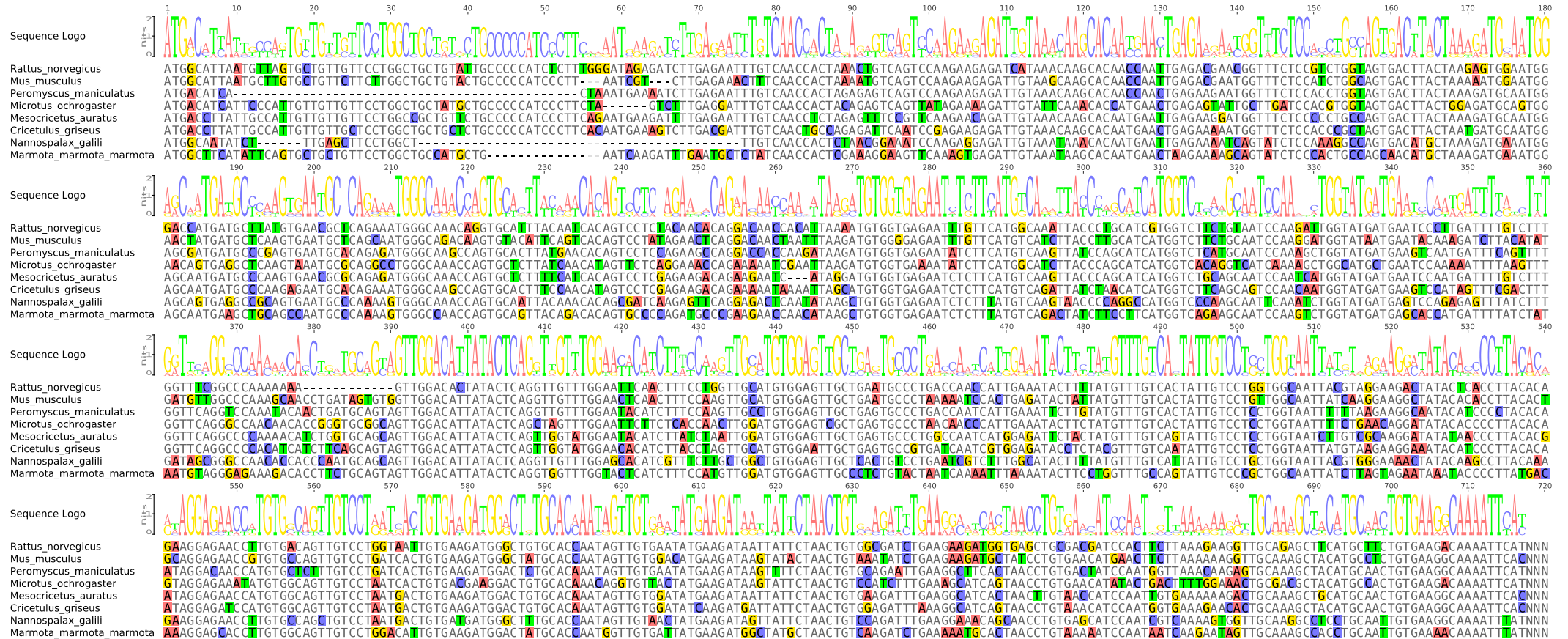

Supplement: Supplementary file 6 — Additional file 6. Crisp3 (Evac3a) gene sequence alignment. [file 12862_2020_1632_MOESM6_ESM.pdf]
