# Supplementary material for: Evolutionary analysis of genes coding for Cysteine-RIch Secretory Proteins (CRISPs) in mammals
Source: BMC Evol Biol. 2020 Jun 8;20:67. doi: 10.1186/s12862-020-01632-5 (PMC7278046; doi:10.1186/s12862-020-01632-5)
Supplement: Supplementary file 3 — Additional file 3. Phenotype and gene sequence accession data. [file 12862_2020_1632_MOESM3_ESM.docx]

| **Additonal file 3. Phenotype and gene sequence accession data.** | | |  |  |  |
| --- | --- | --- | --- | --- | --- |
| **Gene** | **species** | **Body mass** | **Testes mass** | **References** | **Sequence ID** |
| ***Evac 1*** | *Ailuropoda melanoleuca* | 111700.000 | 330.000 | Howard et al 2006 | 100463544 |
|  | *Aotus nancymaae* |  |  |  | 105709578 |
|  | *Balaenoptera scammoni* | 12000000.000 | 8796.000 | McLeod 2010 | 102999792 |
|  | *Bison bison bison* | 731400.000 | 523.600 | Helbig et al 2007 | 105005154 |
|  | *Bos mutus* |  |  |  | 102269022 |
|  | *Bos taurus* | 680385.000 | 681.000 | Kenagy & Trombulak 1986 | 616774 |
|  | *Bubalus bubalis* | 680000.000 | 652.000 | Pant et al 2003 | 102394742 |
|  | *Callithrix jacchus* | 320.000 | 1.300 | Kenagy & Trombulak 1986 | 100395304 |
|  | *Camelus bactrianus* | 816000.000 | 95.000 | Tayeb 1951; Gage 1998 | 105083921 |
|  | *Camelus dromedarius* | 800000.000 | 121.700 | Anderson et al 2004 | 105096584 |
|  | *Camelus ferus* |  |  |  | 102512319 |
|  | *Canis lupus familiaris* | 21620.000 | 27.660 | Woodall & Johnstne 1988 | 100855553 |
|  | *Capra hircus* | 25420.000 | 156.800 | Anderson et al 2004 | 100860898 |
|  | *Cercocebus atys* |  |  |  | 105590597 |
|  | *Chlorocebus sabaeus* |  |  |  | 103221382 |
|  | *Chrysochloris asiatica* |  |  |  | 102827748 |
|  | *Colobus angolensis palliatus* |  |  |  | 105524841 |
|  | *Condylura cristata* | 48.500 | 2.100 | Bedford et al 1999 | 101623146 |
|  | *Cricetulus griseus* | 33.724 | 1.784 | Matsushima et al 1990 | 100756652 |
|  | *Dipodomys ordii* | 56.300 | 0.530 | Ramm 2007 | 105987717 |
|  | *Eptesicus fuscus* |  |  |  | 103296659 |
|  | *Equus caballus* | 468000.000 | 416.000 | Kenagy & Trombulak 1986 | 100033953 |
|  | *Equus przewalskii* |  |  |  | 103540097 |
|  | *Erinaceus europaeus* | 1100.000 | 5.500 | Bedford et al 2004 | 103127288 |
|  | *Felis catus* | 3100.000 | 2.340 | França & Godinho 2003 | 101082060 |
|  | *Gorilla gorilla gorilla* | 134000.000 | 23.200 | Kenagy & Trombulak 1986 | 101135775 |
|  | *Homo sapiens* | 63540.000 | 50.200 | Kenagy & Trombulak 1986 | 167 |
|  | *Ictidomys tridecemlineatus* |  |  |  | 101964505 |
|  | *Leptonychotes weddellii* |  |  |  | 102726237 |
|  | *Macaca fascicularis* | 4787.000 | 35.700 | Kenagy & Trombulak 1986 | 102126120 |
|  | *Macaca mulatta* | 10430.000 | 76.000 | Kenagy & Trombulak 1986 | 574111 |
|  | *Macaca nemestrina* | 9980.000 | 66.700 | Kenagy & Trombulak 1986 | 105490888 |
|  | *Mandrillus leucophaeus* | 28500.000 | 27.300 | Anderson et al 2004 | 105535106 |
|  | *Mesocricetus auratus* | 124.995 | 3.505 | Kenagy & Trombulak 1986 | 101829885 |
|  | *Microtus ochrogaster* | 45.400 | 0.520 | Pierce et al 1990 | 102002729 |
|  | *Mus musculus* | 21.852 | 0.139 | Gómez Montoto et al 2011a | 78081 |
|  | *Mustela putorius furo* | 1047.000 | 3.280 | Kenagy & Trombulak 1986 | 101676389 |
|  | *Myotis brandtii* |  |  |  | 102256361 |
|  | *Myotis davidii* |  |  |  | 102765404 |
|  | *Myotis lucifugus* | 6.800 | 0.108 | Hosken 1997 | 102434934 |
|  | *Nannospalax galili* |  |  |  | 103727373 |
|  | *Nomascus leucogenys* |  |  |  | 100592806 |
|  | *Odobenus rosmarus divergens* | 1233000.000 | 496.200 | Fitzpatrick et al 2012 | 101371756 |
|  | *Oryctolagus cuniculus* | 2888.000 | 6.060 | Kenagy & Trombulak 1986 | 100359052 |
|  | *Otolemur garnettii* |  |  |  | 100965791 |
|  | *Ovis aries* | 57172.730 | 222.991 | Anderson et al 2004 | 101102939 |
|  | *Pan paniscus* | 39100.000 | 135.200 | Dixson & Anderson 2004 | 100985429 |
|  | *Panthera tigris altaica* | 340500.000 | 24.400 | Anderson et al 2004 | 102955288 |
|  | *Pantholops hodgsonii* |  |  |  | 102340286 |
|  | *Pan troglodytes* | 44340.000 | 118.800 | Kenagy & Trombulak 1986 | 740481 |
|  | *Papio anubis* | 26400.000 | 78.420 | Dixson & Anderson 2004 | 101023133 |
|  | *Pongo abelii* |  |  |  | 100435024 |
|  | *Rattus norvegicus* | 379.626 | 3.058 | Kenagy & Trombulak 1986 | 654517 |
|  | *Saimiri boliviensis* |  |  |  | 101030794 |
|  | *Sus scrofa* | 39700.000 | 128.200 | Almeida et al 2006 | 396812 |
|  | *Tarsius syrichta* |  |  |  | 103267565 |
|  | *Trichechus manatus latirostris* |  |  |  | 101361051 |
|  | *Tupaia belangeri* | 141.000 | 1.490 | Zhang et al 2012 | ENSTBET00000013439 |
|  | *Tupaia chinensis* |  |  |  | 102502651 |
|  | *Ursus maritimus* | 427000.000 | 131.400 | Iossa et al 2008 | 103664015 |
|  | *Vicugna pacos* |  |  |  | 102538730 |
| ***Evac 2*** | *Aotus nancymaae* |  |  |  | 105709561 |
|  | *Balaenoptera scammoni* | 12000000.000 | 8796.000 | McLeod 2010 | 102999239 |
|  | *Bos mutus* |  |  |  | 102268180 |
|  | *Bos taurus* | 680385.000 | 681.000 | Kenagy & Trombulak 1986 | 512443 |
|  | *Bubalus bubalis* | 680000.000 | 652.000 | Pant et al 2003 | 102393780 |
|  | *Callithrix jacchus* | 320.000 | 1.300 | Kenagy & Trombulak 1986 | 100414255 |
|  | *Camelus bactrianus* | 816000.000 | 95.000 | Tayeb 1951; Gage 1998 | 105083857 |
|  | *Camelus dromedarius* | 800000.000 | 121.700 | Anderson et al 2004 | 105096576 |
|  | *Camelus ferus* |  |  |  | 102523539 |
|  | *Canis lupus familiaris* | 21620.000 | 27.660 | Woodall & Johnstne 1988 | 474932 |
|  | *Capra hircus* | 25420.000 | 156.800 | Anderson et al 2004 | 100860897 |
|  | *Cavia porcellus* | 813.300 | 4.100 | Kenagy & Trombulak 1986 | 100135505 |
|  | *Cercocebus atys* |  |  |  | 105590635 |
|  | *Chinchilla lanigera* | 422.300 | 5.200 | Kenagy & Trombulak 1986 | 102006008 |
|  | *Chlorocebus sabaeus* |  |  |  | 103221386 |
|  | *Chrysochloris asiatica* |  |  |  | 102811857 |
|  | *Colobus angolensis palliatus* |  |  |  | 105524844 |
|  | *Condylura cristata* | 48.500 | 2.100 | Bedford et al 1999 | 101622458 |
|  | *Cricetulus griseus* | 33.724 | 1.784 | Matsushima et al 1990 | 100764099 |
|  | *Dasypus novemcinctus* | 3850.000 | 8.800 | Torres et al 1983 | 101418102 |
|  | *Echinops telfairi* |  |  |  | 101645773 |
|  | *Elephantulus edwardii* |  |  |  | 102855837 |
|  | *Eptesicus fuscus* | 15.500 |  |  | 103296596 |
|  | *Equus asinus* | 290000.000 | 202.340 | Anderson et al 2004 | ENSECAG00000019933 |
|  | *Equus caballus* | 468000.000 | 416.000 | Kenagy & Trombulak 1986 | 100034138 |
|  | *Equus przewalskii* |  |  |  | 103563017 |
|  | *Felis catus* | 3100.000 | 2.340 | França & Godinho 2003 | ENSFCAG00000028647 |
|  | *Gorilla gorilla* | 134000.000 | 23.200 | Kenagy & Trombulak 1986 | ENSGGOG00000024118 |
|  | *Heterocephalus glaber* | 32.800 | 0.035 | Faulkes et al 1994 | 101697420 |
|  | *Homo sapiens* | 63540.000 | 50.200 | Kenagy & Trombulak 1986 | 7180 |
|  | *Ictidomys tridecemlineatus* |  |  |  | 101965978 |
|  | *Lipotes vexillifer* |  |  |  | 103081206 |
|  | *Loxodonta africana* | 4365500.000 | 4530.000 | Kenagy & Trombulak 1986 | 100677691 |
|  | *Macaca fascicularis* | 4787.000 | 35.700 | Kenagy & Trombulak 1986 | 101925241 |
|  | *Macaca mulatta* | 10430.000 | 76.000 | Kenagy & Trombulak 1986 | 706747 |
|  | *Macaca nemestrina* | 9980.000 | 66.700 | Kenagy & Trombulak 1986 | 105490892 |
|  | *Mandrillus leucophaeus* | 28500.000 | 27.300 | Anderson et al 2004 | 105535110 |
|  | *Mus musculus* | 21.852 | 0.139 | Gómez Montoto et al 2011a | 22024 |
|  | *Mustela putorius furo* | 1047.000 | 3.280 | Kenagy & Trombulak 1986 | 101677276 |
|  | *Myotis brandtii* |  |  |  | 102254851 |
|  | *Myotis davidii* |  |  |  | 102761321 |
|  | *Nomascus leucogenys* |  |  |  | 100594507 |
|  | *Ochotona princeps* |  |  |  | 101530215 |
|  | *Octodon degus* | 233.300 | 1.820 | Goel et al 1998 | 101577929 |
|  | *Orcinus orca* | 7365000.000 | 10311.000 | McLeod 2010 | 101270859 |
|  | *Oryctolagus cuniculus* | 2888.000 | 6.060 | Kenagy & Trombulak 1986 | 100358266 |
|  | *Otolemur garnettii* |  |  |  | 100966430 |
|  | *Ovis aries* | 57172.730 | 222.991 | Anderson et al 2004 | 100462690 |
|  | *Pan paniscus* | 39100.000 | 135.200 | Dixson & Anderson 2004 | 100986879 |
|  | *Panthera tigris altaica* | 340500.000 | 24.400 | Anderson et al 2004 | 102954145 |
|  | *Pantholops hodgsonii* |  |  |  | 102323641 |
|  | *Pan troglodytes* | 44340.000 | 118.800 | Kenagy & Trombulak 1986 | 462756 |
|  | *Papio anubis* | 26400.000 | 78.420 | Dixson & Anderson 2004 | 101020670 |
|  | *Physeter catodon* | 37069874.000 | 12010.639 | McLeod 2010 | 102979550 |
|  | *Pongo abelii* |  |  |  | ENSPPYT00000019413 |
|  | *Propithecus coquereli* |  |  |  | 105826663 |
|  | *Pteropus alecto* | 800.000 | 5.100 | Hosken 1998 | 102879818 |
|  | *Pteropus vampyrus* |  |  |  | ENSPVAG00000016072 |
|  | *Rattus norvegicus* | 379.626 | 3.058 | Kenagy & Trombulak 1986 | 360445 |
|  | *Rhinopithecus roxellana* |  |  |  | 104671493 |
|  | *Saimiri boliviensis* |  |  |  | 101029197 |
|  | *Sorex araneus* | 10.900 | 0.170 | Parapanov et al 2009 | 101555366 |
|  | *Sus scrofa* | 39700.000 | 128.200 | Almeida et al 2006 | 397369 |
|  | *Tarsius syrichta* |  |  |  | 103273618 |
|  | *Tupaia chinensis* |  |  |  | 102487807 |
| ***Evac 3a*** | *Cricetulus griseus* | 33.724 | 1.784 | Matsushima et al 1990 | XM_007646140.1 |
|  | *Marmota marmota marmota* |  |  |  | XM_015487380.1 |
|  | *Mesocricetus auratus* | 124.995 | 3.505 | Kenagy & Trombulak 1986 | XM_005072289.2 |
|  | *Microtus ochrogaster* | 45.400 | 0.520 | Pierce et al 1990 | XM_005372188.1 |
|  | *Mus musculus* | 21.852 | 0.139 | Gómez Montoto et al 2011a | 11571 |
|  | *Nannospalax galili* |  |  |  | XM_008825234.1 |
|  | *Peromyscus maniculatus* | 19.000 | 0.266 | Trainor et al 2006 | XM_016006318.1 |
|  | *Rattus norvegicus* | 379.626 | 3.058 | Kenagy & Trombulak 1986 | 64827 |

**References**

Almeida FFL, Leal MC, França LR (2006) Testis Morphometry, Duration of Spermatogenesis, and Spermatogenic Efficiency in the Wild Boar (Sus scrofa scrofa)1. Biol Reprod. 75:792-799.

Bedford JM, Mock OB, Nagdas SK, Winfrey VP, Olson GE (1999) Reproductive features of the eastern mole (Scalopus aquaticus) and star-nose mole (Condylura cristata). J Reprod Fertil. 117:345-353.

Dixson AF, Anderson MJ (2004) Sexual behavior, reproductive physiology and sperm competition in male mammals. Physiol Behav 83:361–371.

Faulkes CG, Trowell SN, Jarvis JUM, Bennett NC (1994) Investigation of numbers and motility of spermatozoa in reproductively active and socially suppressed males of two eusocial African mole-rats, the naked mole-rat (Heterocephalus glaber) and the Damaraland mole-rat (Cryptomys damarensis). J Reprod Fertil 100:411-416

Fitzpatrick JL, Almbro M, Gonzalez‐Voyer A, Kolm N, Simmons LW (2012) Male contest competition and the coevolution of weaponry and testes in pinnipeds. Evolution, 66(11), 3595-3604.

França LR, Godinho CL 2003) Testis Morphometry, Seminiferous Epithelium Cycle Length, and Daily Sperm Production in Domestic Cats (Felis catus). Biol Reprod. 68:1554-1561.

Goel N, Lee TM, Pieper DR (1998) Removal of the olfactory bulbs delays photic reentrainment of circadian activity rhythms and modifies the reproductive axis in male Octodon degus. Brain Res. 792:229-236.

Gómez Montoto L, Magaña C, Tourmente M, Martín-Coello J, Crespo C, Luque-Larena JJ, Gomendio M, Roldan ERS (2011) Sperm competition, sperm numbers and sperm quality in muroid rodents. Plos One. 6:e18173.

Helbig L, Woodbury MR, Haigh JC, Collins J, Barth, AD (2007) The seasonal fertility of North American bison (Bison bison) bulls. Animal reproduction science, 97(3), 265-277.

Hosken DJ (1997) Sperm competition in bats. Proc R Soc Lond B. 264:385-392.

Hosken DJ (1998) Testes mass in megachiropteran bats varies in accordance with sperm competition theory. Behav Ecol Sociobiol. 44:169-177.

Howard J, Zhang Z, Li D, Huang Y, Hou R, Li G, Zhang M, Ye Z, Zhang J, Huang S, Spindler R, Zhang H, Wildt DE (2006) Male reproductive biology in giant pandas in breeding programmes in China. In: Wildt DE, Zhang A, Zhang H, Janssen DL, Ellis S (eds) Giant Panda Biology Medicine and Management. Cambridge University Press, Cambridge, UK, pp 159-197

Iossa G, Soulsbury CD, Baker PJ, Harris S (2008) Sperm competition and the evolution of testes size in terrestrial mammalian carnivores. Func Ecol. 22:655-662.

Kenagy GJ, Trombulak C (1986) Size and function of mammalian testes in relation to body size. J Mammal 67:1–22.

Matsushima S, Sakai Y, Hira Y (1990) Effect of photoperiod on pineal gland volume and pinealocyte size in the Chinese hamster, Cricetulus griseus. American journal of anatomy, 187(1), 32-38.

MacLeod CD (2010) The relationship between body mass and relative investment in testes mass in cetaceans: Implications for inferring interspecific variations in the extent of sperm competition. Marine Mammal Science. 26:370-380.

Pant HC, Sharma RK, Patel SH, Shukla HR, Mittal AK, Kasiraj R, Misra AK, Prabhakar JH (2003) Testicular development and its relationship to semen production in Murrah buffalo bulls. Theriogenology 60:27-34

Parapanov RN, Nusslé S, Crausaz M, Senn A, Hausser J, Vogel P (2009) Testis size, sperm characteristics and testosterone concentrations in four species of shrews (Mammalia, Soricidae). Animal reproduction science, 114(1), 269-278.

Pierce JD, Ferguson B, Salo AL, Sawrey DK, Shapiro LE, Taylor SA, Dewsbury DA. 1990. Patterns of sperm allocation across successive ejaculates in four species of voles (Microtus). J Reprod Fertil. 88:141-149.

Ramm SA (2007) Sexual selection and genital evolution in mammals: A phylogenetic analysis of baculum length. Am Nat. 169:360-369.

Gage MJG (1998) Mammalian sperm morphometry. Proc R Soc Lond B. 265:97-103.

Torres CN, Godinho HP, Machado ABM. (1983) Seasonal variation in spermatogenesis in the nine-banded armadillo (Dasypus novemcinctus) from Southeastern Brazil. Anim Reprod Sci. 6:135-141.

Trainor BC, Martin LB 2nd, Greiwe KM, Kuhlman JR, Nelson RJ (2006) Social and photoperiod effects on reproduction in five species of Peromyscus. General and comparative endocrinology 148:252-259.

Woodall PF, Johnstone IP (1988) Dimensions and allometry of testes, epididymides and spermatozoa in the domestic dog (Canis familiaris). J Reprod Fertil 82:603-609

Zhang YX, Ping SH, Yang SH (2012) Morphological characteristics and cryodamage of Chinese tree shrew (Tupaia belangeri chinensis) sperm.
