# Supplementary material for: Evolutionary analysis of genes coding for Cysteine-RIch Secretory Proteins (CRISPs) in mammals
Source: BMC Evol Biol. 2020 Jun 8;20:67. doi: 10.1186/s12862-020-01632-5 (PMC7278046; doi:10.1186/s12862-020-01632-5)
Supplement: Supplementary file 7 — Additional file 7. Phylogenetic trees of species included in the analysis for A-Evac1 and B-Evac2. [file 12862_2020_1632_MOESM7_ESM.pdf]

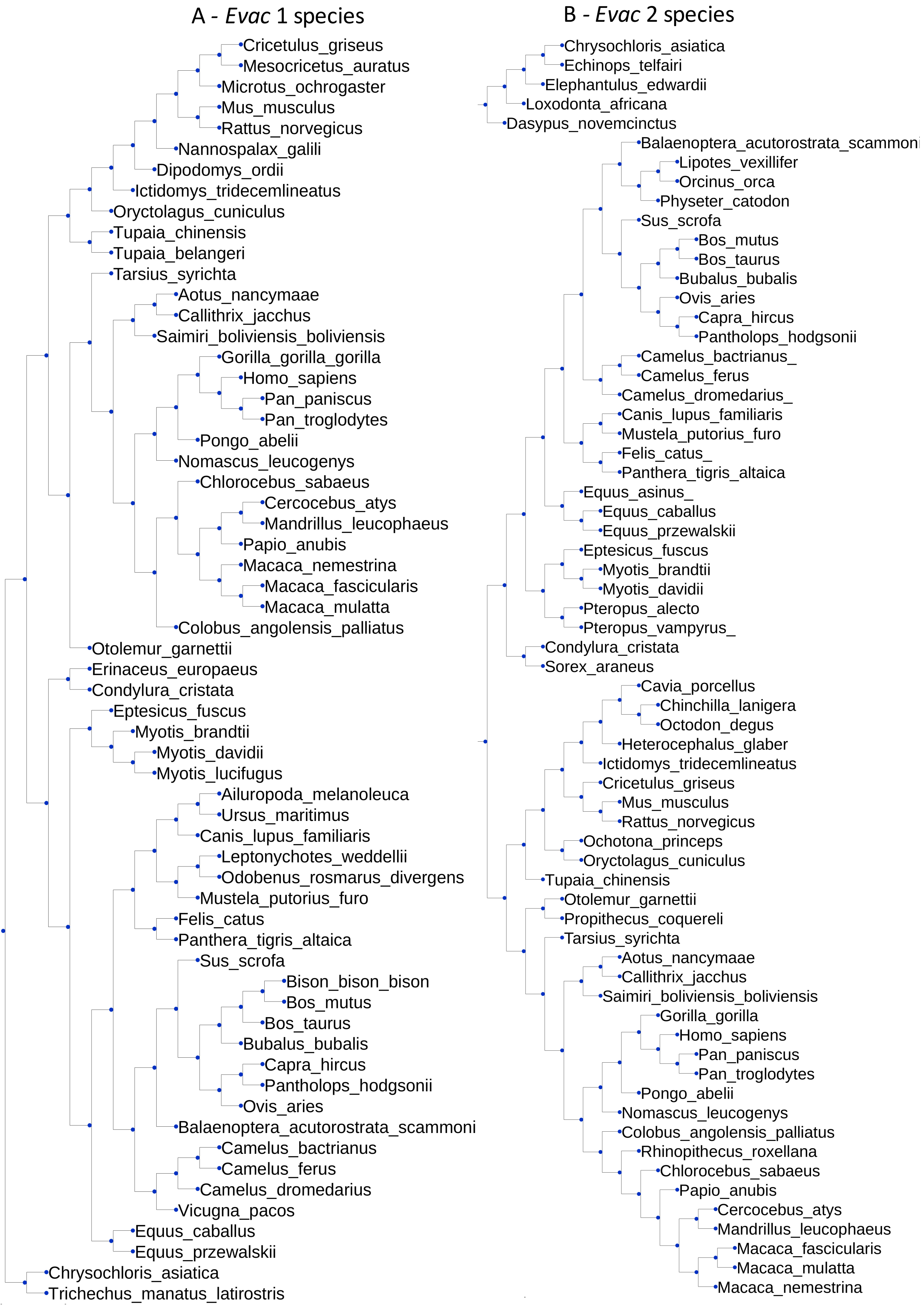

Additional file 7. Phylogenetic trees of species included in the analysis for A - *Evac*1 and B - *Evac*2

Evac 3 species

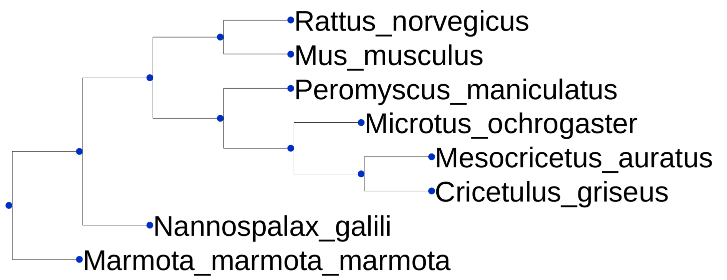

Additional file 8. Phylogenetic trees of species included in the analysis for *Evac3*

References for additional files 7 and 8

Adkins RM, Walton AH, Honeycutt RL (2003) Higher-level systematics of rodents and divergence time estimates based on two congruent nuclear genes. Mol Phylogenet Evol26:409–420.

Agnarsson I, May-Collado LJ (2008) The phylogeny of Cetartiodactyla: The importance of dense taxon sampling, missing data, and the remarkable promise of cytochrome b toprovide reliable species-level phylogenies. Mol Phylogenet Evol 48:964–985.

Agnarsson I, Zambrana-Torrelio CM, Flores-Saldana NP, May-Collado LJ (2011) A time-calibrated species-level phylogeny of bats (Chiroptera, Mammalia). PLOS Currents Tree of Life. 2011 Feb 4. Edition 1

Almeida FC, Bonvicino CR, Cordeiro-Estrela P (2007). Phylogeny and temporal diversification of Calomys (Rodentia, Sigmodontinae): Implications for the biogeographyof an endemic genus of the open/dry biomes of South America. Mol Phylogenet Evol42:449–466.

Álvarez A, Perez SI, Verzi DH (2011) Ecological and phylogenetic influence on mandible shape variation of South American caviomorph rodents (Rodentia: Hystricomorpha). Biol. J. Linn. Soc. 102:828-837.

Baena A, Mootnick AR, Falvo JV, Tsytskova AV, Ligeiro F, Diop OM, Brieva C, GagneuxP, O'Brien SJ, Ryder OA, Goldfeld AE (2005) Primate TNF promoters reveal markers ofphylogeny and evolution of innate immunity. PLoS One 2:e621.

Beck RM, Bininda-Emonds OR, Cardillo M, Liu FR, Purvis A (2006) A higher-level MRP supertree of placental mammals. BMC Evol Biol 6:93–107.

Bininda-Emonds OR, Cardillo M, Jones KE, MacPhee RDE, Beck RMD, Greyner R, PriceSA, Vos RA, Gittleman JL, Purvis A (2007) The delayed rise of present-day mammals.Nature 446:507–512.

Bininda-Emonds ORP, Cardillo M, Jones KE, MacPhee RDE, Beck RMD, Grenyer R, Price SA, Vos RA, Gittleman JL, Purvis A (2007) The delayed rise of present-day mammals. Nature 446:507-512.

Böhm M, Mayhew PJ (2005) Historical biogeography and the evolution of the latitudinal gradient of species richness in the Papionini (Primata: Cercopithecidae). Biol J Linn Soc-Lond 85:235–246.

Borges BN, Paiva TS, Harada ML (2008) Evolution of the SEC1 gene in New World monkey lineages (Primates, Platyrrhini). Genet Mol Res 7:663–678.

Bradley RD, Durish ND, Rogers DS, Miller JR, Engstrom MD, Kilpatrick CW (2007) Toward a molecular phylogeny for Peromyscus: evidence from mitochondrial cytochrome-b sequences. J Mammal 88:1146–1159.

Conroy CJ, Cook JA (2000) Molecular systematics of a holartic rodent (Microtus: Muridae). J Mammal 81:344–359.

Fabre PH, Hautier L, Dimitrov D, Douzery EJP (2012) A glimpse on the pattern of rodent diversification: a phylogenetic approach. BMC Evol Biol 12:88.

Flynn JJ, Finarelli JA, Zehr S, Hsu J, Nedbal MA (2005) Molecular phylogeny of the Carnivora (Mammalia): assessing the impact of increased sampling on resolving enigmaticrelationships. Syst Biol 54:317–337.

Gomendio M, Tourmente M, Roldan ERS (2011) Why mammalian lineages respond differently to sexual selection: metabolic rate constrains the evolution of sperm size. Proc. R. Soc. Lond. B 278:3135-3141.
