# Supplementary material for: Evolutionary analysis of genes coding for Cysteine-RIch Secretory Proteins (CRISPs) in mammals
Source: BMC Evol Biol. 2020 Jun 8;20:67. doi: 10.1186/s12862-020-01632-5 (PMC7278046; doi:10.1186/s12862-020-01632-5)
Supplement: Supplementary file 2 — Additional file 2. PhylomeDB tree of seeding on mouse CRISP3 (EVAC4). [file 12862_2020_1632_MOESM2_ESM.pdf]

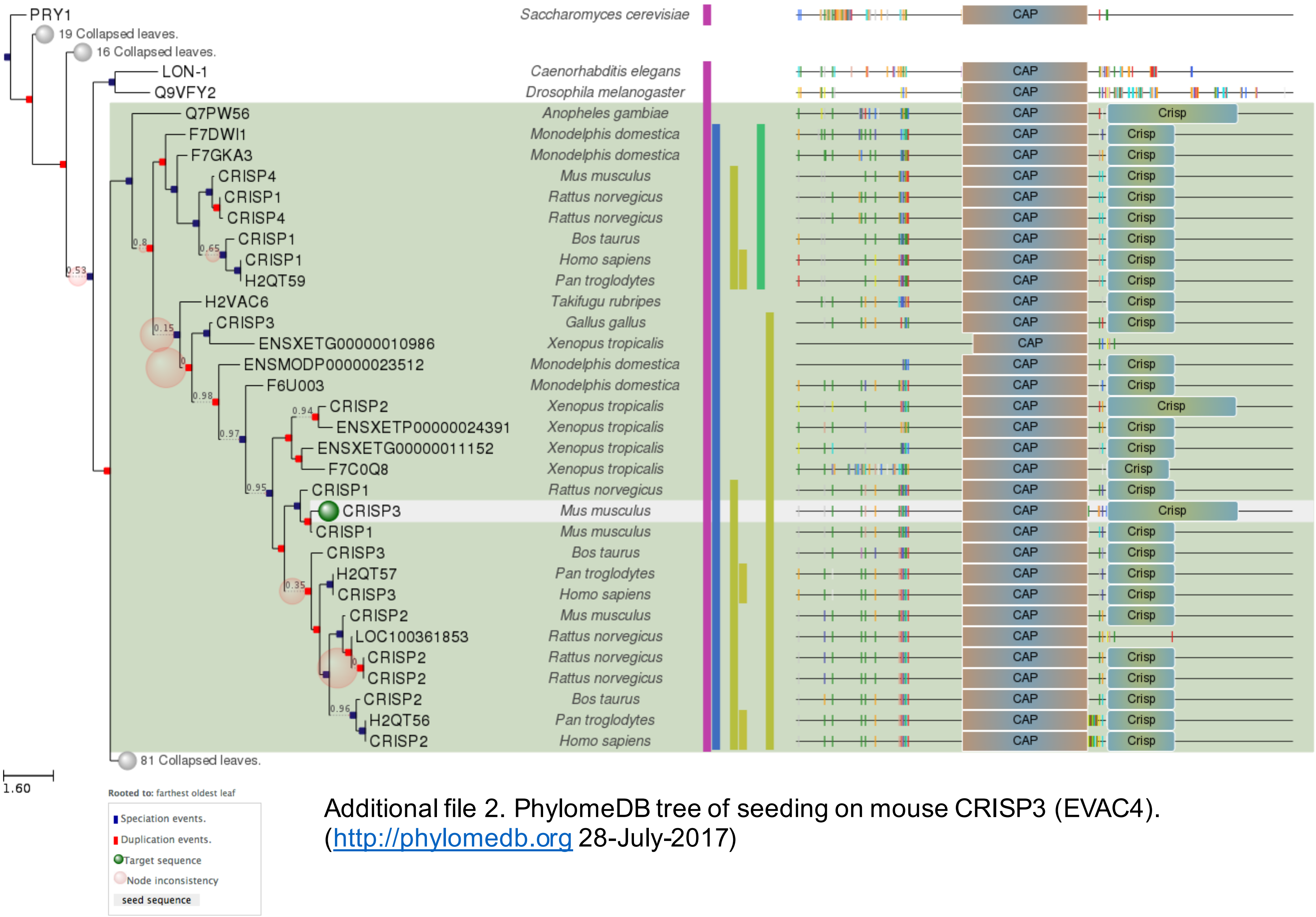

Additional file 2. PhylomeDB tree of seeding on mouse CRISP3 (EVAC4). (http://phylomedb.org 28-July-2017)
